# Cisplatin-selected resistance is associated with increased motility and stem-like properties via activation of STAT3/Snail axis in atypical teratoid/rhabdoid tumor cells

**DOI:** 10.18632/oncotarget.2737

**Published:** 2015-01-31

**Authors:** Wei-Hsiu Liu, Ming-Teh Chen, Mong-Lien Wang, Yi-Yen Lee, Guang-Yuh Chiou, Chian-Shiu Chien, Pin-I Huang, Yi-Wei Chen, Ming-Chao Huang, Shih-Hwa Chiou, Yang-Hsin Shih, Hsin-I Ma

**Affiliations:** ^1^ Graduate Institute of Medical Sciences, National Defense Medical Center, Taipei, Taiwan; ^2^ Department of Neurological Surgery, Tri-Service General Hospital and National Defense Medical Center, Taipei, Taiwan; ^3^ School of Medicine, National Yang-Ming University, Taipei, Taiwan; ^4^ Department of Neurosurgery, Neurological Institute, Taipei Veterans General Hospital & National Yang-Ming University, Taipei, Taiwan; ^5^ Institute of Clinical Medicine, National Yang-Ming University, Taipei, Taiwan; ^6^ College of Biological Science and Technology, National Chiao Tung Univeristy, Taiwan; ^7^ Division of Pediatric Neurosurgery, Department of Neurosurgery, Neurological Institute, Taipei Veterans General Hospital, Taipei, Taiwan; ^8^ Cancer Center, Taipei Veterans General Hospital, Taipei, Taiwan; ^9^ Department of Medical Research and Education, Taipei Veterans General Hospital, Taiwan

**Keywords:** Atypical teratoid/rhabdoid tumor (ATRT), STAT3, Snail, oncogenic resistance and cisplatin

## Abstract

Atypical teratoid/rhabdoid tumor (ATRT) is a malignant pediatric brain tumor with great recurrence after complete surgery and chemotherapy. Here, we demonstrate that cisplatin treatment selects not only for resistance but also for a more oncogenic phenotype characterized by high self-renewal and invasive capabilities. These phenomena are likely due to STAT3 upregulatoin which occurred simultaneously with higher expression of Snail, an activator of epithelial–mesenchymal transition (EMT), in ATRT-CisR cells. STAT3 knockdown effectively suppressed Snail expression and blocked motility and invasion in ATRT-CisR cells, while overexpressing Snail reversed these effects. Chromatin immunoprecipitation assay indicated that STAT3 directly bound to Snail promoter. Moreover, STAT3 knockdown effectively suppressed cancer stem-like properties, synergistically enhanced the chemotherapeutic effect, and significantly improved survival rate in ATRT-CisR-transplanted immunocompromised mice. Finally, immunohistochemistrical analysis showed that STAT3 and Snail were coexpressed at high levels in recurrent ATRT tissues. Thus, the STAT3/Snail pathway plays an important role in oncogenic resistance, rendering cells not only drug-resistant but also increasingly oncogenic (invasion, EMT and recurrence). Therefore, the STAT3/Snail could be a target for ATRT treatment.

## INTRODUCTION

Atypical teratoid/rhabdoid tumor (ATRT) is a rare, fatal pediatric tumor of the central nervous system (CNS) that typically occurs in patients under 3 years of age [1]. Patients with ATRT have a poor prognosis and a short survival that ranges from approximately 16 to 24 months [2, 3]. ATRT contains a unique heterogeneous combination of cells, including rhabdoid cells and peripheral epithelial and mesenchymal elements. Due to the histological similarity on magnetic resonance imaging (MRI) of ATRT and medulloblastoma, ATRT is easily misclassified as a primitive neuroectodermal tumor/medulloblastoma [1, 4]. Radiation is not a treatment option for ATRT patients because neuroradiation causes significant neurocognitive deficiency in children or infants with ATRT [5]. The standard therapeutic treatment for ATRT patients involves surgical resection and chemotherapy, which slightly improves the relatively low disease-free survival outcome [2, 3]. Although brain tumors rarely metastasize to distant organs, ATRT displays aggressive behavior that promotes tumor dissemination, including intracranial and spinal cord invasion [6].

Cisplatin (cis-diamminedichloroplatinum; CDDP)-based chemotherapy regimens are the standard treatment for ATRT patients who have received surgical resection [7]. However, during the course of chemotherapy, resistance to chemotherapeutic drugs often develops and causes the tumor to recur. High recurrence rates and drug-resistance potential have been reported in ATRT even after completion of combined therapy [8, 9]. It has been reported that chemotherapy can increase oncogenic phenotype, such as invasiveness and cancer stem cell (CSC)-derived self-renewal capabilities [10]. Moreover, oncogenic resistance, associated with activation of pathways of cell proliferation and suppression of apoptosis, confers resistance to the growth-inhibitory carcinogenic environment, eventually causes to the aggressive manners of cancer malignancy [11]. For example, Bcr-Abl, a fusion of Bcr-Abl that results in the constitutively active kinase Bcr–Abl, renders cells resistant to apoptosis caused by DNA damaging drugs and simultaneously renders a cell leukemogenic [12]. Furthermore, chemotherapy-induced tumor progression, a type of selection for oncogenic resistance, renders cells resistant to chemotherapy and simultaneously promotes its oncogenic potential [11]. However, the interplay between cisplatin-selected resistance and ATRT recurrence is still unclear.

The epithelial-mesenchymal transition (EMT) in cancer induces a triad of cancer features: invasion and metastasis; stem cell properties; and drug resistance [13]. In hepatomas, activation of EMT and hedgehog signaling are associated with chemoresistance and invasion [14]. Certain EMT transcription factors, such as Snail and Slug, have been reported to induce radioresistance and chemoresistance by antagonizing p53-mediated apoptosis [15] and directly contribute to cisplatin resistance in ovarian cancer [16]. ZEB1, another regulator of EMT, has been shown to influence invasion, chemoresistance and tumorigenesis, and orchestrate key features of a CSC-like phenotype in malignant gliomas [13]. Vice versa, drug resistance in cancer cells has been implicated in the positive regulation of EMT. It has been reported that cisplatin-resistant lung cancer cells acquire an EMT-like phenotype and CSC-like properties through the AKT/β-catenin/Snail signaling pathway [17]. Furthermore, cisplatin treatment of primary and metastatic epithelial ovarian carcinomas generates residual cells with a mesenchymal stem cell-like profile [18]. Recently, Sun and colleagues showed evidence that chemotherapy-induced EMT in human tongue cancer cells occurs through Bmi-1 targeting by miR-200b and miR-15b [19]. Cisplatin also induces resistance to molecular-targeted agents through an activated EMT pathway [20]. However, the function of EMT and aoosiated processes in CNS cancer has received little attention so far. It is believed that critical invasion pathways/EMT-like properties overlap between CNS and other cancers [13, 21]. Nevertheless, the interplay between EMT-like pathways and chemoresistance in ATRT has not yet been clarified.

Signal-transducer-and-activator-of-transcription 3 (STAT3), a transcription factor involved in cytokine signaling, participates in the regulation of cell cycle, apoptosis, cell invasion, and angiogenesis [22]. Recent studies have shown that STAT3 activation in brain tumors, such as gliomas and medulloblastomas, is a prognostic indicator for malignant progression, tumor growth, and a low patient survival rate [23]. STAT3 is also involved in regulating the EMT process in several cancer types [24, 25]. STAT3 activation is required for TGF-β-induced EMT in lung cancer cells [26]. Through cooperation with EGFR, STAT3 upregulates Twist and subsequently induces EMT [27]. Moreover, the JAK/STAT3/Snail signaling pathway activates head and neck tumor metastasis and EMT [28]. Additionally, an IL-6/STAT3/miR34a feedback loop has been shown to promote EMT-mediated cancer invasion and metastasis in human colorectal cancer cells [29]. The role of STAT3 in regulating chemoresistance has recently been emphasized in cancer cells such as glioblastoma multiforme (GBM) and neuroblastoma. Activation of the IL6/STAT3 pathway protects GBM and neuroblastoma cells from drug-induced apoptosis [30, 31]. In breast cancer cells, an autocrine signaling between STAT3 and RANTES is essential for tamoxifen resistance [32]. Targeting of the STAT3 protein was shown to effectively kill GBM cells and suppress GBM tumor growth [33]. However, it remains unknown whether STAT3 signaling is involved in the acquisition of chemosensitivity and in enhancing EMT-related properties with tumor invasion in ATRT.

In our study, we found that cisplatin-selected resistant ATRT (CisR; ATRT-CisR) cells displayed higher STAT3 expression and it plays a role in oncogenic phenotype, such as cell motility, tumor invasion, and chemoresistance. Chromatin immunoprecipitation (ChIP) assay in ATRT-CisR cells confirmed that STAT3 directly bound to the promoter of Snail, which is known to induce EMT. We showed that STAT3/Snail signaling played an important role in oncogenic resistance, rendering cells not only to be drug-resistant but also oncogenic (invasion, EMT and recurrence). That result suggested that this signaling pathway is a novel treatment target for ATRT patients. Our findings indicate interplay between chemoresistance and tumor invasion/EMT-like properties in ATRT.

## RESULTS

### Increased tumor invasion and upregulated STAT3 in cisplatin-selected resistant ATRT cells

**Figure 1:**
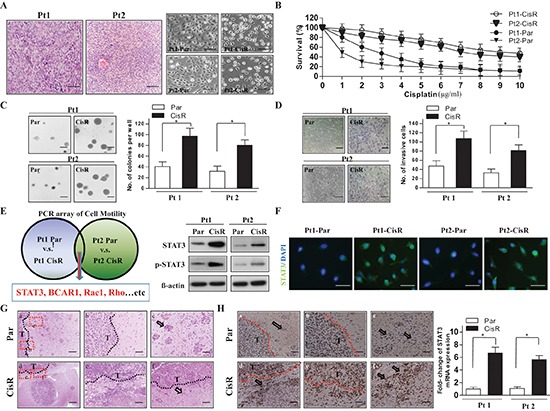
Cisplatin-selected resistant ATRT cells display a more malignant phenotype and increased expression of STAT3 **(A)** Left: Representative clinicopathological picture of ATRT. The tissue sample was stained with H&E staining. Scale bars, 50 μm. Right: ATRT-parental (ATRT-Par) cells display a round, lymphoid-like appearance and grow in tight clusters with substantial cellular cohesion. Comparatively, ATRT-cisplatin resistant (ATRT-CisR) cells have a more spindle-like morphology and display decreased intercellular contacts. Moreover, ATRT-CisR cells extend pseudopodia. Scale bars, 50 μm. **(B)** Dose-response curves were generated for all four cell lines, termed Pt1-Par, Pt2-Par, Pt1-CisR, and Pt2-CisR, and the IC50 concentrations were calculated as 3 μg/ml, 1 μg/ml, 8 μg/ml, and 9 μg/ml, respectively. **(C–D)** ATRT-Par and ATRT-CisR cells in two individual patients were subjected to soft agar colony formation (C), and invasion (D) assays to assess the ATRT phenotype. Scale bars, 50 μm. *P < 0.01 by Student's *t*-test. **(E)** Left: Schema for identifying the motility-related genes of STAT3, BCAR1, Rac1, and Rho by Human Cell Motility RT^2^ Profiler PCR Array. Right: Western blot analysis of STAT3 in ATRT-Par and ATRT-CisR cells of two individual patients. **(F)** CisR cells showed a higher expression of STAT3 than Par cells by immunofluorescence staining. The nuclei were visualized with DAPI staining. Scale bars, 20 μm. **(G)** Patterns of invasion of ATRT-Par and ATRT-CisR in mouse brains. H&E staining showing infiltration of the brain parenchyma by cells detached from the main tumor mass in mice inoculated with ATRT-Par and ATRT-CisR cells. Upper panel: ATRT-Par cells show low invasive characteristics of clear tumor boundary (b) and large tumor islands (c; arrow) including a stellate appearance. Lower panel: ATRT-CisR cells have invasive characteristics of non-clear tumor boundary (e) and small islands (f; arrow) with single cell invasion and invasion as clusters of cells along the white matter tracts. Scale bars, 200 μm (a and d), and 100 μm (b, c, d and e). **(H)** Brain specimens isolated from ATRT-CisR cells show higher STAT3 expression levels than those isolated from ATRT-Par cells as determined through IHC staining (left) and qPCR analysis (right). Scale bars, 100 μm (a and d), and 50 μm (b, c, d and e). *P < 0.01 by Student's *t*-test. T: main tumor mass. The data shown are the mean ± SD of three independent experiments.

The role of cisplatin as a chemotherapy drug in modulating or generating the aggressive character of ATRT, and causing relapse and resistance to conventional therapeutics is still unclear. To undertand this, we established two ATRT primary cell lines from two patients (Pt1 and Pt2; Figure 1A, left). The two ATRT patients' samples exhibited morphological features of celluar tumor with small round cells, rhabdoid cells and prominent nucleolus by Hematoxylin and Eosin (H&E) staining. The cisplatin IC50 values in Pt1 and Pt2 cells were determined to be approximately 3 μg/ml and 1 μg/ml, respectively. These concentrations of cisplatin were then used to treat Pt1 and Pt2 cells for 3 months to establish corresponding age- and passage-matched cisplatin-selected resistant cell lines (Pt1-CisR and Pt2-CisR).

Previous studies have shown that CisR cancer cell lines have a characteristic fibroblastic morphology and reduced intercellular contacts [16]. We also found that the Pt1-CisR and Pt2-CisR cells displayed a spindle-like morphology and formed a non-cohesive sheet compared with the round lymphoid-like parental cells (Pt1-Par and Pt2-Par; Figure 1A, right). To determine the difference in cisplatin IC50 values between the established parental and resistant ATRT cells, a cisplatin dose-response curve for treatments ranging from 1 to 10 μg/ml was determined for the cells. The cell survival curve showed significantly different IC50 values between parental and resistant ATRT cells (Figure 1B; Pt1-Par, 3 μg/ml; Pt2-Par, 1 μg/ml versus Pt1-Cis, 9 μg/ml; Pt2-CisR, 8 μg/ml). Furthermore, ATRT-CisR cells showed an enhanced ability for anchorage-independent growth on agarose (Figure 1C) as well as increased migration (Supplementary Figure 1A) and invasion (Figure 1D) compared with their parental cells.

To further investigate potential downstream pathways of cisplatin-induced cell motility, we compared the gene expression levels of cell motility-associated factors using RT^2^ Profiler PCR arrays in the two pairs of parental and resistant cells. We focused on cell motility –related genes and selected those genes that were more strongly expressed (>1.5 fold) in ATRT-CisR than ATRT-Par cells. The results revealed that BCAR1, Rac1, Rho, and STAT3 were increased expression in ATRT-CisR cells compared with ATRT-Par cells (Figure 1E, left). The mRNA and protein levels of these molecules were confirmed by qPCR and Western blot analysis, respectively. STAT3 displayed the most significant difference between ATRT-Par and ATRT-CisR cells (Figure 1E, right; Supplementary Figure 1B–Figure 1C). Immunofluorescent staining also confirmed higher STAT3 expression in ATRT-CisR cells than in ATRT-Par cells (Figure 1F). To analyze the invasive characteristics of ATRT-Par and ATRT-CisR cells, the two cell lines were injected into SCID mice. Examination of paraffin sections of xenograft tumors from dissected brains showed that ATRT-Par tumors had low invasive characteristics, including a clear tumor boundary, large tumor islands, and a stellate appearance. In contrast, ATRT-CisR tumors showed significant invasive morphology including small islands with single-cell invasion (Figure 1G). Importantly, specimens from ATRT-CisR tumors had higher expression levels of STAT3 than those from ATRT-Par tumors, as shown by immunohistochemistry (IHC) staining (Figure 1H, left) and qPCR analysis (Figure 1H, right). These data support our observation that cisplatin resistance increases tumorigenicity and that STAT3 expression is associated with cell motility and invasiveness in cisplatin-selected resistant ATRT cells.

### STAT3 promotes tumor invasion through induction of EMT factors

**Figure 2:**
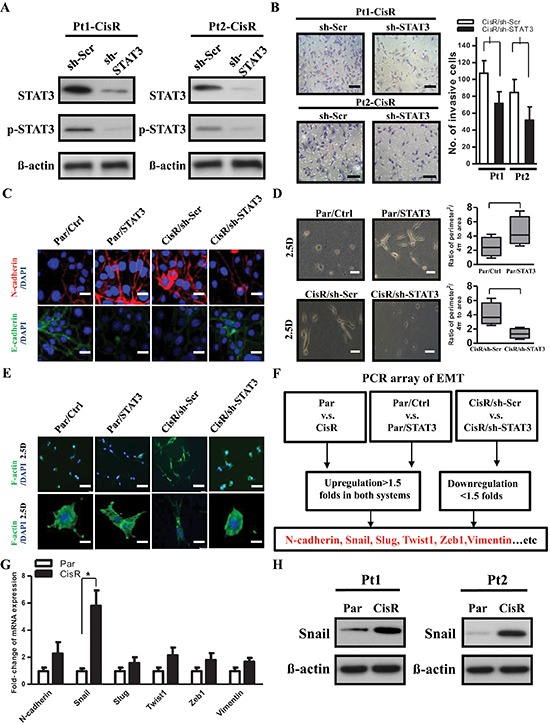
EMT activator is downstream of STAT3-induced tumor invasion **(A)** Stable cell lines with STAT3 knockdown were generated in previously established Pt1-CisR and Pt2-CisR cells. The expression or knockdown efficiency of STAT3 was analyzed by Western blot. **(B)** Pt1-CisR cells transfected with the scrambled shRNA control vector (Pt1-CisR/sh-Scr), Pt1-CisR cells transfected with the sh-STAT3 vector (Pt1-CisR/sh-STAT3), Pt2-CisR cells transfected with the scrambled shRNA control vector (Pt2-CisR/sh-Scr), and Pt2-CisR cells transfected with the sh-STAT3 vector (Pt2-CisR/sh-STAT3) were subjected to an invasion assay. Scale bars, 50 μm. *P < 0.01 by Student's *t*-test. **(C)** Expression of N-cadherin and E-cadherin were analyzed in ATRT-Par cells that were transfected with the STAT3-overexpressing vector (Par/STAT3) or the vector control (Par/Ctrl) and ATRT-CisR cells transfected with the sh-STAT3 vector (CisR/sh-STAT3) or the scrambled shRNA control vector (CisR/sh-Scr) by immunofluorescence staining. The nuclei were visualized with DAPI staining. Scale bars, 5 μm. **(D)** Phase-contrast images of Par/Ctrl, Par/STAT3, CisR/sh-Scr and CisR/sh-STAT3 (*n* = 200 for each stable cell line). The cells were cultivated on top of thick collagen (2.5D). Scale bars, 20 μm. *P < 0.01 by Student's *t*-test. **(E)** Immunofluorescence micrographs showing the morphology and actin organization of Par/Ctrl, Par/STAT3, CisR/sh-Scr and CisR/sh-STAT3. The cells were cultured in 2.5D. Green, F-actin; blue, nuclei. Scale bars, 50 μm (first row) and 5 μm (second row). **(F)** Schema for identifying EMT-related genes by Human Epithelial to Mesenchymal Transition (EMT) RT^2^ Profiler PCR Array. **(G)** A qPCR analysis of EMT-related genes N-cadherin, Snail, Slug, Twist1, Zeb1 and Vimentin. *P < 0.01 by Student's *t*-test. **(H)** Western blot of the target gene Snail. The data shown are the mean ± SD of three independent experiments.

The correlation between STAT3 expression level and ATRT invasiveness prompted us to investigate whether STAT3 regulates the invasive properties of ATRT-CisR cells. We knocked down STAT3 expression in the two ATRT-CisR cell lines using short hairpin RNA (shRNA) constructs (Figure 2A). STAT3-knockdown cells were subjected to transwell migration and invasion assays, which revealed that STAT3 knockdown dramatically suppressed cell motility and invasiveness in ATRT-CisR cells (Figure 2B and Supplementary Figure 2A). Previous studies suggested that EMT might promote tumor invasion, increase chemoresistance and activate cancer stem-like capacities [13]. Here, we found that STAT3-overexpressed ATRT-Par cells (Par/STAT3) and scrambled shRNA control-transfected ATRT-CisR cells (CisR/sh-Scr) had a higher level of N-cadherin and a lower level of E-cadherin. Conversely, empty vector-transfected ATRT-Par (Par/Ctrl) and STAT3-knockdown ATRT-CisR (CisR/sh-STAT3) cells had lower N-cadherin levels and higher E-cadherin levels as assessed by both immunofluorescence staining and Western blot (Figure 2C and Supplementary Figure 2B). Interestingly, when the cells were cultured on top of thick collagen (to establish 2.5D culture conditions), Par/STAT3 and CisR/sh-Scr cells exhibited an elongated morphology, while Par/Ctrl and CisR/sh-STAT3 cells had rounded shapes (Figure 2D). This finding implied that in ATRT-CisR cells, STAT3 may modulate cell morphology and mesenchymal movement in 2.5D. Moreover, in the 2.5D-cultivated system, Par/STAT3 and CisR/sh-Scr cells exhibited pseudopod protrusions, whereas Par/Ctrl and CisR/sh-STAT3 exhibited cortical actin arrangement (Figure 2E). Taken together, our data support a role for STAT3 in activating tumor invasion and enhancing the EMT-like phenotype in cisplatin-selected resistant ATRT cells.

The association between STAT3 expression levels, cell motility, and an EMT-like phenotype in ATRT cells suggested that STAT3 may regulate ATRT invasive properties through an EMT mechanism. To investigate the potential downstream targets of STAT3-mediated tumor invasion, we performed RT^2^ Profiler PCR arrays for gene expression levels of EMT associated factors with three pairs of cells: ATRT-Par vs. ATRT-CisR; Par/Ctrl vs. Par/STAT3; and CisR/sh-Scr vs. CisR/sh-STAT3. We focused on EMT-related genes and selected those genes that were more strongly expressed (>1.5 fold) in ATRT-CisR, Par/STAT3, and CisR/sh-Scr cells than in their counterparts (Figure 2F). The PCR arrays results were confirmed by qPCR and Western blot in ATRT-Par and ATRT-CisR cells and revealed that Snail displayed the most significant difference between ATRT-Par and ATRT-CisR cells (Figure 2G, Figure 2H; Supplementary Figure 2C).

### The STAT3/Snail axis is critical for EMT-like phenotype and tumor invasion in cisplatin-selected resistant ATRT cells

**Figure 3:**
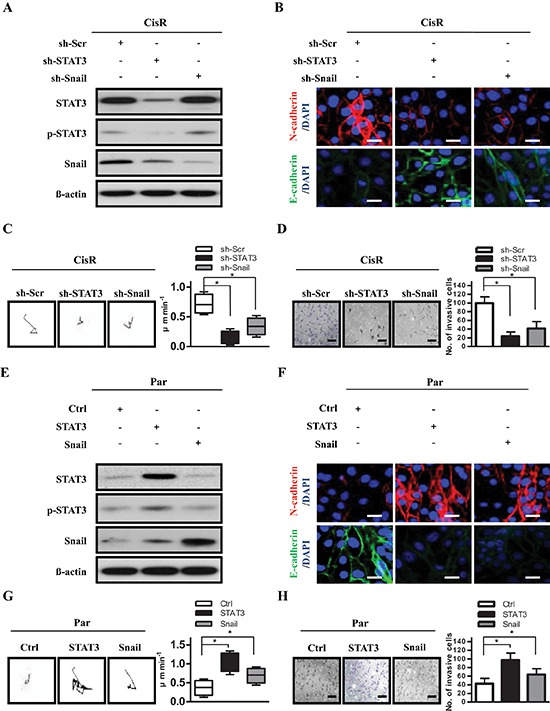
STAT3 activates cell motility and tumor invasion through Snail **(A)** Expression of STAT3, phosphorylated STAT3 (p-STAT3), and Snail in ATRT-CisR cells. ß-actin was used as a loading control. **(B)** Expression of N-cadherin and E-cadherin were examined in CisR/sh-Scr, CisR/sh-STAT3 and CisR/sh-Snail cells by immunofluorescence staining. The nuclei were visualized with DAPI staining. Scale bars, 5 μm. **(C)** Representative trajectories and quantification of speed in ATRT-CisR cells transfected with sh-STAT3 or sh-Snail versus scrambled shRNA control vector (sh-Scr; *n* = 10 for each group). *P < 0.01 by Student's *t*-test. **(D)** Transwell invasion assay in ATRT-CisR cells transfected with sh-STAT3 or sh-Snail versus scrambled shRNA control vector (sh-Scr). Scale bars, 50 μm. *P < 0.01 by Student's *t*-test. **(E)** Expression of STAT3, p-STAT3, and Snail in ATRT-Par cells. β-actin was used as a loading control. **(F)** Expression of N-cadherin and E-cadherin were analyzed in Par/Ctrl, Par/STAT3, and Par/Snail cells by immunofluorescence staining. The nuclei were visualized with DAPI staining. Scale bars, 5 μm. **(G)** Representative trajectories and quantification of speed in ATRT-Par cells transfected with ectopic STAT3 or Snail versus the vector control (Ctrl; *n* = 10 for each group). *P < 0.01 by Student's *t*-test. **(H)** Transwell invasion assay in ATRT-Par cells transfected with ectopic STAT3 or Snail versus the vector control (Ctrl). Scale bars, 50 μm. *P < 0.01 by Student's *t*-test. The data shown are the mean ± SD of three independent experiments.

The increase of Snail in invasive ATRT-CisR cells led us to speculate that Snail may be a modulator of STAT3-mediated EMT-like phenotypic cell motility and invasion. We examined the causal link between STAT3 and Snail, and found that knockdown of endogenous STAT3 downregulated Snail expression, but not vice versa (Figure 3A). Knockdown of STAT3 or Snail decreased the expression of N-cadherin and increased the expression of E-cadherin, by immunofluorescence staining (Figure 3B). In time-lapse microscopy, the low-STAT3 and low-Snail ATRT-CisR cells had rounded shapes and displayed substantially decreased motility in the 2.5D environment (Figure 3C). Cells with knockdown of STAT3 or Snail had reduced invasive ability compared with control cells (Figure 3D). Consistently, ectopic STAT3 expression increased Snail protein expression, while ectopic overexpression of Snail did not affect STAT3 expression or phosphorylation (Figure 3E). Moreover, ectopic STAT3 or Snail enhanced the expression of N-cadherin but attenuated the expression of E-cadherin, based on immunofluorescence staining (Figure 3F). Using time-lapse microscopy, we found more rapid movement in the elongated high-STAT3 and high-Snail ATRT-CisR cells in the 2.5D environment (Figure 3G). Moreover, cells overexpressing STAT3 or Snail had higher invasive abilities than control cells (Figure 3H). Taken together, these results indicate that STAT3 upregulates Snail and contributes to tumor invasion and motility in ATRT cells.

### STAT3 directly regulates Snail transcription

**Figure 4:**
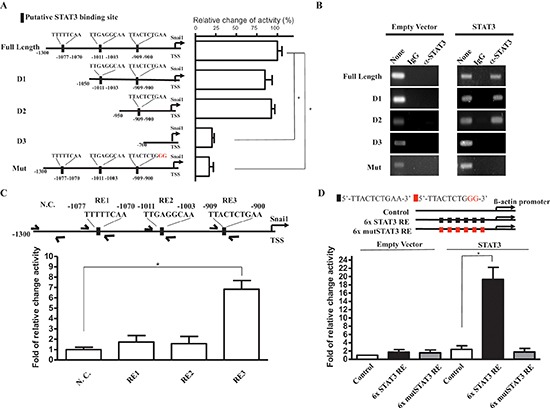
STAT3 activates Snail promoter by STAT3 responsive sequence **(A)** Left: A graphical illustration of the constructed full-length, deleted, and mutated of Snail promoter-driven reporter plasmids. Right: a quantitation of luciferase activity driven by STAT3 on indicated promoter on the graphical illustration in ATRT-Par cells. **(B)** ChIP analysis of STAT3 at full length and mutated Snail promoter region. Secific STAT3-binding signal of ectopic expression of STAT3 compared with control (empty vector) in ATRT-Par cells. IgG antibody was used as a negative control. **(C)** Q-ChIP analysis of STAT3 at indicated loci on Snail promoter, including nonspecific control (N.C), RE1, RE2, and RE3 in ATRT-CisR cells. Input, 2% of total lysate. **(D)** Quantitation of luciferase activity driven by ectopic expression of STAT3 on 6x STAT3 RE and 6x Mut STAT3 RE constructed β-actin promoter in ATRT-CisR cells. The data shown are the mean ± SD of three independent experiments.

Because STAT3 is a critical transcription factor for modulating malignancies among many different cancers, we next aimed to elucidate whether STAT3 enhances Snail expression through transcriptional regulation. We screened for potential STAT3 binding sites in a 1.3-Kb upstream sequence from the Snail transcriptional start site and found three potential binding regions (RE): RE1, 5′-TTTTTCAA-3′ (–1077 to –1070); RE2, 5′-TTGAGGCAA-3′ (–1011 to –1003); and RE3: 5′-TTACTCTGAA-3′ (–909 to –900). To determine whether STAT3 activates Snail expression is a promoter sequence dependent, we constructed a series of Snail promoter-driven luciferase reporter plasmids with a full-length promoter, promoter regions with different lengths of deletions (D1-3), or a promoter with mutations in the potentially candidated binding sites (Mut; Figure 4A, left). We then cotransfected a STAT3 expression vector with the serial deletion constructs in ATRT cells as indicated. The reporter assays demonstrated that RE3 was responsible for STAT3-mediated promoter activity, suggesting that RE3 is the STAT3 binding site (Figure 4A, right).

To further characterize the Snail promoter sequence dependencies of STAT3 to the activations of Snail promoter, we performed a ChIP analysis for a complementary assessment of promoter activity. ATRT-Par cells were co-transfected with empty vector or ectopic STAT3 expression vector combined with firefly luciferase reporting vectors with various Snail promoter regions. Specific STAT3-binding signals were detected with full length, D1 and D2 Snail promoter vectors but not with D3 and mutated Snail promoter vectors, indicating that the binding of STAT3 on the Snail promoter is dependent on the RE3 sequence within the Snail promoter region (Figure 4B). To further confirm the STAT3 binding site in the endogenous Snail promoter, and whether the binding of STAT3 reflects the actual endogenous Snail promoter activations, we performed endogenous Q-ChIP with primers specific to the Snail promoter REs (RE1-3) in ATRT-CisR cells. The results, consistent with our reporter and ChIP assays (Figure 4C), indicate STAT3 activates endogenous Snail promoter activities through the same targeting sequences as previous exogenous co-transfection experiment results (Input, 2% of total lysate). To further confirm the specific Snail promoter activation by STAT3 targeting sequences, we next generated reporter vectors with either 6 repeats of the putative binding sequence concluded by previous reporter and ChIP experiment results, 5′-TTACTCTGAA-3′, or 6 repeats of a mutated binding sequence, 5′-TTACTCTGGG-3, upstream of ß-actin minimal promoter in ATRT-CisR cells. The reporter assay results showed that the binding sequence 5′-TTACTCTGAA-3′, responsed to STAT3 overexpresions by 20-folds of increase as compared with respective control experiments, while 6 repeats of mutated sequence were unable to response to STAT3 induced transcriptional activation. (Figure 4D). Taken together, we conclude that STAT3 directly regulates Snail transcription through specific binding to the sequence 5′-TTACTCTGAA-3′ in region RE3 of the Snail promoter exogenously and endogenously. The STAT3/Snail transcriptional regulator axis is the key for aberrant malignancies of ATRT cells in our experimental systems.

### The STAT3/Snail axis regulates cancer stem-like and tumor-initiating properties

Previous studies revealed that drug resistance is usually enhanced in cancer stem-like cells [34, 35]. The increased cisplatin resistance in our established ATRT-CisR cells raised the potential of these cells to be cancer stem-like cells. Comparative analysis between ATRT-Par and ATRT-CisR cells showed that ATRT-CisR cells have higher sphere-forming ability (Supplementary Figure 3A) and expressed higher levels of stemness factors such as Oct-4, Nanog, Sox2, Bmi-1, and Nestin (Supplementary Figure 3B). Flow cytometry also showed that the expression level of CD133 was dramatically increased in ATRT-CisR cells (Supplementary Figure 3C).

**Figure 5:**
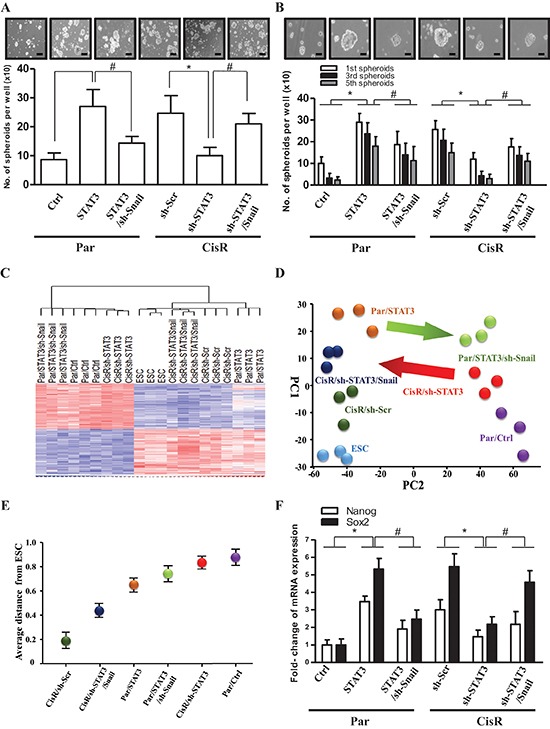
The STAT3/Snail axis induces stemness and tumor-initiating ability in ATRT-CisR cells **(A)** Sphere-forming and **(B)** self-renewal assays revealed sphere-forming frequency in Par/Ctrl, Par/STAT3, Par/STAT3 cells transfected with the sh-Snail vector (Par/STAT3/sh-Snail), CisR/sh-Scr, CisR/sh-STAT3, and CisR/sh-STAT3 transfected with the Snail-overexpressing vector (CisR/sh-STAT3/Snail). Scale bars, 50 μm. *P < 0.01; ^#^*P* < 0.01 by Student's *t*-test. **(C)** Gene expression microarray analysis (gene tree) were differentially expressed in human embryonic stem cell (ESC), ATRT-Par/Ctrl cells, ATRT-Par/STAT3 cells, ATRT-Par/STAT3/sh-Snail cells, ATRT-CisR/sh-Scr cells, ATRT-CisR/sh-STAT3 cells, and ATRT-CisR/sh-STAT3/Snail cells as demonstrated by a hierarchical heat map. **(D)** Principle component analysis (PCA) and **(E)** Multidimensional scaling (MDS) analysis revealed the average lineage transcriptome distances between ESCs and ATRT-Par/Ctrl, ATRT-Par/STAT3, ATRT-Par/STAT3/sh-Snail ATRT-CisR/sh-Scr, ATRT-CisR/sh-STAT3, and ATRT-CisR/sh-STAT3/Snail cells. **(F)** A qPCR analysis of Nanog and Sox2 in Par/Ctrl, Par/STAT3, Par/STAT3/sh-Snail, CisR/sh-Scr, CisR/sh-STAT3, and CisR/sh-STAT3/Snail cells. *P < 0.01; ^#^*P* < 0.01 by Student's *t*-test. Data shown are the mean ± SD of three independent experiments.

As STAT3 and Snail are regulators of self-renewal and cancer stem-like properties in several solid tissue cancers [36], we hypothesized that the STAT3/Snail axis activates cancer stem-like and tumor-initiating properties in ATRT cells. We first investigated the involvement of STAT3 and Snail in cancer stem-like properties and tumor-initiating capability of ATRT-CisR cells using sphere-forming and self-renewal assays. We observed that STAT3 overexpression in ATRT-Par cells increased sphere numbers through several passages of the sphere-formation assay, indicating an increased self-renewal capability, whereas simultaneous knockdown of Snail attenuated the STAT3-increased self-renewal (Figure 5A–Figure 5B). Consistently, co-overexpression of Snail rescued cells from inhibited self-renewal mediated by STAT3 knockdown (Figure 5A–5B).

We next examined the stemness transcriptome profile by gene expression microarray analysis (Figure 5C). Principle component analysis (PCA) and multidimensional scaling (MDS) analysis demonstrated that suppression of STAT3 in ATRT-CisR cells diverted them away from ESCs; however, Snail co-overexpression induced cells toward ESCs (Figure 5D–Figure 5E). Consistently, STAT3 overexpression in ATRT-Par cells promoted a signature shift toward that of ESCs, whereas co-knockdown of Snail diverted ATRT-Par cells from ESCs (Figure 5D–Figure 5E). According to bioinformatics data, we hypothesized that the STAT3/Snail axis positively regulates cancer stem-like properties and tumor-initiating capabilities in ATRT. We further analyzed Nanog and Sox2 mRNA expression and showed that STAT3 knockdown in ATRT-CisR cells significantly decreased mRNA levels of Nanog and Sox2, while they were increased after STAT3 overexpression in ATRT-Par cells (Figure 5F). Importantly, orthotopic grafts of the established ATRT clones in mice demonstrated that Snail overexpression rescued the tumor-initiating capability of CisR/sh-STAT3 cells, and Snail knockdown partially reduced the tumor-initiating capability of Par/STAT3 cells (Table 2). Taken together, we conclude that STAT3 and Snail are both essential for promoting tumor-initiating capabilities in ATRT cancer cells and play a key role in chemoresistance-induced cancer stem-like properties of ATRT.

### Blocking STAT3/Snail axis upregulates ABCC1 expression and improves chemoresistance *in vitro*

**Figure 6:**
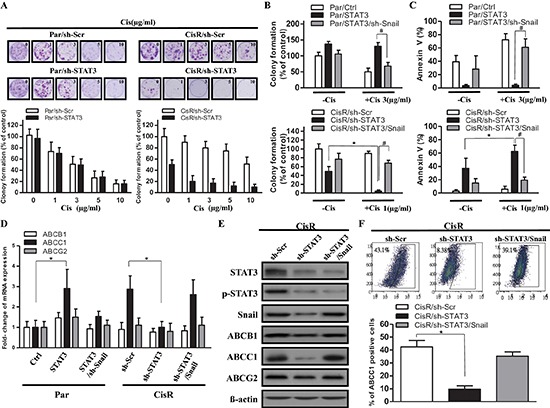
Decreasing STAT3/Snail signaling combined with cisplatin suppresses cell viability and upregulates ABCC1 expression **(A)** To determine the effect of chemotherapy on the tumor survival fraction, a cisplatin dose ranging from 0 to 10 μg/ml was used to treat ATRT-CisR cells with sh-Scr or sh-STAT3 and ATRT-Par cells with sh-Scr or sh-STAT3. **(B)** Colony formation and **(C)** AnnexinV staining assays were performed in ATRT-Par cells treated with vector control (Ctrl), ectopic expression of STAT3 (STAT3), or ectopic expression of STAT3 transfected with sh-Snail (STAT3/sh-Snail) combined with or without cisplatin (1 μg/ml); in addition, ATRT-CisR cells were treated with scrambled shRNA control vector (sh-Scr), sh-STAT3, or sh-STAT3/Snail combined with or without cisplatin (3 μg/ml). *P < 0.01; ^#^*P* < 0.01 by Student's *t*-test. **(D)** A qPCR analysis of ABCB1, ABCC1 and ABCG2 in ATRT-Par cells treated with vector control (Ctrl), STAT3, and STAT3/sh-Snail in addition to ATRT-CisR cells treated with sh-Scr, sh-STAT3, and sh-STAT3/Snail. *P < 0.01 by Student's *t*-test. **(E)** Western blot analysis of ABCB1, ABCC1 and ABCG2 in ATRT-CisR cells treated with sh-Scr, sh-STAT3, or sh-STAT3/Snail. **(F)** The protein expression of ABCC1 in ATRT-CisR cells treated with sh-Scr, sh-STAT3, or sh-STAT3/Snail by flow cytometry. The data shown are the mean ± SD of three independent experiments.

Given that STAT3 was initially identified by its upregulation in cisplatin-selected resistant ATRT cells, we aimed to examine its involvement in chemoresistance in ATRT cells. We subjected Par/Ctrl, Par/sh-STAT3, CisR/sh-Scr, and CisR/sh-STAT3 cells derived from patients to a colony-formation assay in the presence of cisplatin ranging from 0 to 10 μg/ml (Pt1 and Pt2 ATRT cells had similar results; Pt1 cells are shown as representative results). Results showed that STAT3 knockdown severely repressed cisplatin resistance in ATRT-CisR cells but not in ATRT-Par cells. This is most likely due to the fact that STAT3 expression is low in ATRT-Par cells, and attempts to knockdown STAT3 did not alter STAT3 levels. The IC50 value of ATRT-Par/sh-Scr and ATRT-Par/sh-STAT3 were approximately 3 μg/ml (Figure 6A, left), and the IC50 value of ATRT-CisR/sh-STAT3 was approximately 1 μg/ml (Figure 6A, right). These IC50 values (3 μg/ml and 1 μg/ml) were used to treat ATRT-Par and ATRT-CisR stable cell lines, respectively, to evaluate the effect of the STAT3/Snail axis in cisplatin resistance. Colony formation was significantly decreased in ATRT-CisR/sh-STAT3 cells when treated with cisplatin (1 μg/ml) compared with nontreated ATRT-CisR/sh-STAT3 cells (Figure 6B, lower panel). This reduction could be rescued by co-overexpression of Snail (Figure 6B, lower panel). In contrast, cisplatin (3 μg/ml) was not able to suppress the colony formation of ATRT-Par cells with STAT3 overexpression, and co-transfection of sh-Snail partially decreased cisplatin resistance in ATRT-Par/STAT3 cells (Figure 6B, upper panel).

Annexin V staining showed that ATRT-CisR/sh-STAT3 cells had increased Annexin V staining, which was further increased by cisplatin treatment (1 μg/ml), while co-overexpression of Snail decreased the percentage of Annexin V-positive cells (Figure 6C, bottom). In contrast, STAT3 overexpression in ATRT-Par cells decreased the percentage of Annexin V-positive cells, and cisplatin treatment had limited ability to increase this percentage, while co-knockdown of Snail dramatically increased staining (Figure 6C, top). Three ATP-binding cassette (ABC) drug transporters, ABCB1 (p-glycoprotein/MDR1), ABCC1 (MRP1) and ABCG2 (BCRP), have been shown to be involved in the development of drug resistance [37]. We examined the mRNA and protein expression levels of ABCB1, ABCC1, and ABCG2 and observed ABCC1 reduction upon STAT3 knockdown in ATRT-CisR cells and an ABCC1 level increase after STAT3 overexpression in ATRT-Par cells (Figure 6D–Figure 6E). Flow cytometry analysis further confirmed the positive correlation between ABCC1 expression and activity of the STAT3/Snail axis in ATRT-CisR cells (Figure 6F). Taken together, these results showed that STAT3/Snail is crucial for the acquisition of cisplatin resistance in ATRT cells and that ABCC1 is a potential downstream target of the STAT3/Snail axis in modulating cisplatin resistance.

### Abrogation of STAT3/Snail axis synergistically improves the efficacy of chemotherapy and prolongs the survival of recipients of ATRT-CisR cells

**Figure 7:**
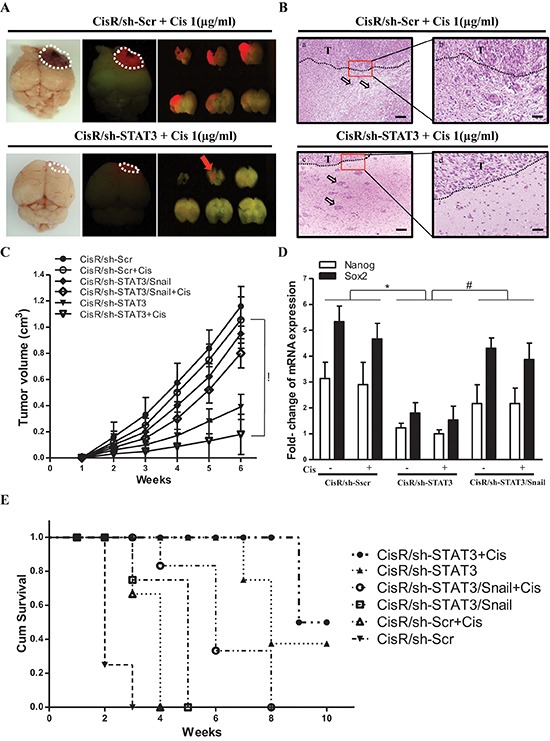
STAT3/Snail axis silencing increases the synergistic effects with chemosensitivity and prolongs the survival of ATRT-CisR *in vivo* ATRT-CisR cells were intracranially transplanted into NOD-SCID mice, with six mice in each group (*n* = 6 in each group; total, 36 mice). **(A)** After 6 weeks, *in vivo* RFP imaging showed that transplanted ATRT-CisR-RFP cells grew solid tumors at the injection site. The tumor volumes in ATRT-CisR/sh-STAT3 cells transplanted mice were significantly decreased treated with cisplatin (1 μg/ml) compared to ATRT-CisR/sh/Scr cells treated with cisplatin (1 μg/ml). **(B)** H&E staining showed paraffin sections of xenograft tumors from dissected brains. Upper panel: ATRT-CisR/sh-Scr tumors treated with cisplatin (1 μg/ml) presented the high invasive characteristics of small islands (a; arrow) with sigle cell invasion and non-clear tumor boundary (b). Lower panel: ATRT-CisR/sh-STAT3 tumors treated with cisplatin (1 μg/ml) presented low invasive characteristics of clear tumor boundary (d), and large tumour islands (c; arrow) including a stellate appearance. Scale bars, 100 μm (a and c), and 50 μm (b and d). T: main tumor mass. **(C)** Tumor volumes in ATRT-CisR transplanted mice treated with sh-STAT3 combined with cisplatin (1 μg/ml) treatment were significantly smaller than those receiving the different protocol. *P < 0.01 by Student's *t*-test. **(D)** A qPCR analysis of Nanog, and Sox2 in CisR/sh-Scr, CisR/sh-STAT3, and CisR/sh-STAT3/Snail cells with or without cisplatin in transplanted mice. *P < 0.01 by Student's *t*-test. **(E)** Kaplan-Meier survival analysis further described mean survival rate for animals injected with ATRT-CisR cells treated with indicated treatments. Mice with ATRT-CisR cells treated with shSTAT3 and cisplatin had a significantly prolonged survival rate compared with untreated ATRT-CisR mice. *P < 0.01 by Student's *t*-test. The data shown are the mean ± SD of three independent experiments.

We further investigated the role of the STAT3/Snail signaling and its effects on cisplatin treatment in ATRT-Par and ATRT-CisR cells *in vivo*. ATRT-CisR cells were labeled with red fluorescent protein (RFP) by lentiviral infection with a vector containing the RFP gene. A total of 1 × 10^5^ ATRT-Par and ATRT-CisR cells with different treatment protocols were injected into the stratum of SCID mice, and tumor size was monitored by 3T MRI for 6 weeks. RFP imaging revealed that tumor volumes in mice transplanted with ATRT-CisR/sh-STAT3 cells were significantly decreased with cisplatin (1 μg/ml) compared to ATRT-CisR/sh-Scr cells with cisplatin (1 μg/ml) (Figure 7A). Examination of paraffin sections of xenograft tumors from dissected brains showed that ATRT-CisR/sh-STAT3 tumors treated with cisplatin (1 μg/ml) had low invasive characteristics, including large tumor islands and a clear tumor boundary (Figure 7B, bottom), compared to ATRT-CisR/sh-Scr tumors treated with cisplatin (1 μg/ml) (Figure 7B, top). Notably, cisplatin showed a synergistic effect with STAT3 knockdown to significantly reduce tumor volumes in the transplanted mice (Figure 7C).

In contrast, ATRT-Par/Ctrl cells treated with cisplatin showed slow tumor growth in transplanted mice, and ectopic STAT3 effectively activated tumor growth of ATRT-Par/Ctrl cells in the transplanted mice (Supplementary Figure 4A). QPCR analysis confirmed that the level of Nanog and Sox2 were significantly decreased in xenograft tumor sections from ATRT-CisR/sh-STAT3-transplanted mice compared with the levels observed after all other treatments (Figure 7D). Compared with untreated ATRT-CisR mice, sh-STAT3 combined with cisplatin (1 μg/ml) significantly increased the survival rate of mice bearing ATRT-CisR intracranial xenografts (Figure 7E). Moreover, compared with untreated ATRT-Par-transplanted mice, ectopic expression of STAT3 resulted in decreased survival rate of intracranial xenograft-bearing mice (Supplementary Figure 4B). These results indicated that the STAT3/Snail axis regulates drug resistance and cancer stem-like properties in xenotransplanted immunocompromised mice.

### Upregulation of STAT3 and Snail expression in clinical samples of recurrent ATRT

**Figure 8:**
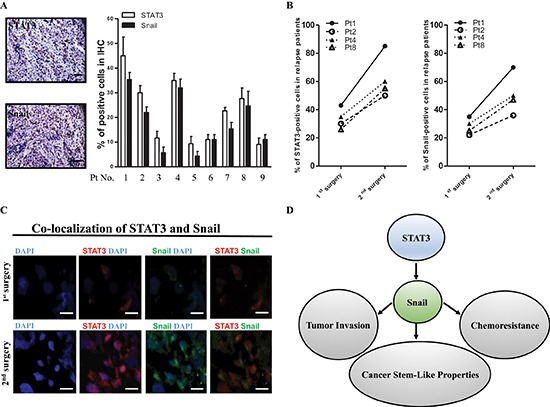
High-level coexpression of STAT3 and Snail present in recurrent ATRT samples **(A)** Detection of STAT3 and Snail protein by IHC staining in 9 ATRT patient samples. Scale bars, 50 μm. **(B)** The percentage of STAT3- and Snail-positive ATRT cells (1st surgery, 9 patients) was dramatically elevated in the tumor-relapse samples (2nd surgery, 4 patients). **(C)** Immunofluorescent staining showing STAT3 and Snail protein colocalization in Pt1's tissues. Scale bars, 20 μm. **(D)** Schematic model depicting the STAT3/Snail axis interconnected between invasion, chemoresistance and tumor initiation.

To confirm the results derived from *in vitro* and animal experiments, we next investigated the levels of STAT3 and Snail by IHC staining in samples from nine ATRT patients. The properties of these patients were noted (Table 1), and representative IHC results are shown in Figure 8A. We observed that the IHC grading of Snail was closely related to STAT3 expression in the nine ATRT patients. As shown in Table 1, eight of the nine patients received full course chemotherapy after their 1^st^ surgery. However, in five patients (patients 1, 2, 4, 7, and 8), the tumor relapsed, and the patients underwent a second surgery. The percentage of STAT3- and Snail-positive cells were dramatically increased in the four of five tumor-relapse samples (patients 1, 2, 4, and 8) compared with the tumor samples from the first surgery (Figure 8B). The results seem to revelaed the levels of STAT3/snail may predict the recurrence of ATRT patients. In support of the closely associated relationship of the two molecules in patient samples, we confirmed the colocalization between STAT3 and Snail in the same foci of ATRT tissue from Pt1 with STAT3^hi^ Snail^hi^ (Figure 8C). In summary, we found that cisplatin-selected resistance (oncogenic resistance) transactivates STAT3/Snail pathway, and the axis regulates tumor migration/invasion, cancer stem-like cell properties, and cisplatin resistance in ATRT cells (Figure 8D).

**Table 1:** ATRT patients' description and characteristics

Patient No.	Age/Sex	Treatment	Survival time
1	2.3 / F	1st Surgery + Chemotherapy + 2nd surgery	0.3 yr
2	8.1 / F	1st Surgery + Chemotherapy + 2nd surgery	4.7 yr
3	0.7 / M	1st Surgery	0.2 yr
4	5.1 / M	1st Surgery + Chemotherapy + 2nd surgery	1.7 yr
5	1.4 / M	1st Surgery + Chemotherapy	8.7 yr
6	3.3 / F	1st Surgery + Chemotherapy	7.5 yr
7	2.8 / M	1st Surgery + Chemotherapy + 2nd surgery	4.4 yr
8	5.1 / M	1st Surgery + Chemotherapy + 2nd surgery	1.7 yr
9	1.7 / M	1st Surgery + CCRT	2.5 yr

The second surgery for tumor relapses.

**Table 2:** STAT3/Snail axis regulated the tumor-initiating activity of ATRT *in vivo*

Patients	Injected Cells Numbers	CisR/sh-Scr	CisR/sh-STAT3	CisR/sh-STAT3 + Snail	Par/Ctrl	Par/STAT3	Par/STAT3 + sh-Snail
No. 1	50,000	3/3	3/3	3/3	3/3	3/3	3/3
	10,000	3/3	2/3	3/3	2/3	3/3	2/3
	1,000	3/3	1/3	3/3	0/3	3/3	2/3
	500	2/3	0/3	1/3	0/3	0/3	0/3
	100	2/3	0/3	1/3	0/3	0/3	0/3
	50	0/3	0/3	0/3	0/3	0/3	0/3
No. 2	50,000	3/3	3/3	3/3	3/3	3/3	3/3
	10,000	3/3	2/3	3/3	2/3	3/3	2/3
	1,000	3/3	2/3	3/3	0/3	3/3	2/3
	500	2/3	1/3	2/3	0/3	1/3	1/3
	100	1/3	0/3	0/3	0/3	0/3	0/3
	50	0/3	0/3	0/3	0/3	0/3	0/3
No. 3	50,000	3/3	3/3	3/3	1/3	3/3	3/3
	10,000	3/3	1/3	3/3	2/3	2/3	1/3
	1,000	2/3	1/3	1/3	0/3	1/3	1/3
	500	0/3	0/3	0/3	0/3	0/3	0/3
	100	0/3	0/3	0/3	0/3	0/3	0/3
	50	0/3	0/3	0/3	0/3	0/3	0/3

Atypical teratoid/rhabdoid tumor- CisR/sh-Scr, CisR/sh-STAT3, CisR/sh-STAT3 + Snail, Par/Ctrl, Par/STAT3 and Par/STAT3 + sh-Snail transfected cells were transplanted into the brain striatum of mice with different number of cells as indicated (*N* = 3). After 12 weeks follow-up, the presence of tumor nodules in each mouse was determined and listed in the table.

## DISCUSSION

ATRT is the most common primary pediatric tumor of the CNS and has limited treatment options and a dismal prognosis. Because of the rarity of ATRT and resistance to therapeutic regimens, no standard chemotherapy regimen has been established [38]. Although cisplatin-based chemotherapy is a mainstay treatment of ATRT, drug resistance frequently develops [39]. Many studies have attributed chemoresistance and tumor recurrence to the existence of a subset of CSCs or cancer-initiating cells that possess stem-like properties and are able to repopulate tumors [19]. Recently, studies have indicated that therapy induced “oncogenic-type” may be involve in self-renewal, cancer invasiveness, drug resistance, and EMT, as well as share the properties of CSCs [11]. In this study, we are able to provide the following findings: (i) expression of STAT3 and Snail is highly correlated in different cancer cell lines; (ii) cisplatin treatment selects not only for resistance but also for a more oncogenic phenotype characterized by high self-renewal and invasive capabilities; (iii) STAT3 directly regulates Snail transcription and activation of STAT3/Snail pathway contributes to tumor invasion, chemoresistance and cancer stem-like properties; (iv) suppression of Snail in STAT3-overexpressing ATRT cells reduced cisplatin resistance; and (v) coexpression of STAT3 and Snail in primary ATRT samples enhanced cisplatin resistance and correlated with worse prognosis. We demonstrated that cisplation can activate “oncogenic-type” of drug resistance and Snail plays a crucial role in STAT3-dependent induction of oncogenic phenotype, such as cisplatin resistance, tumor invasion and cancer stem-like properties. To our knowledge, the present study is the first to show that STAT3 directly regulates Snail transcription and promotes platinum resistance.

It has been reported that oncogenic resistance is associated with highly aggressive cancer phenotype, and modulates the therapeutics-induced cell cycle arrest and apoptosis [11]. For example, hypoxia transactivates genes, such as autocrine or paracrine growth factors, that are critical for invasion and metastasis during the antiangiogenic therapy [40]. Furthermore, some study revealed Bcr-Abl mutation-mediated drug resistance led to activation of Stat3 associated with malignant cell transformation [41]. Moreover, Src confers resistance to Adriamycin that was associated with the interaction of p21waf1 with the STAT3 transcription factor at the Myc promoter [42]. Consistent with these previous reports, our study found that cisplatin can cause “oncogenic-type” of drug resistance which simultaneously activates STAT3/Snail pathway to make cell aggressive, such as tumor invasion and cancer stem-like capability in ATRT-CisR cells. These findings indicated that chemotherapeutic resistance may contribute to adapt the cancer stem-like capability and simultaneously makes cells more aggressive phenotype in ATRT-CisR cells, in partly through activation of STAT3/Snail pathway. Therefore, the more future studies are needed to explore whether oncogenic reistance play an important role in the development of EMT-derived invasiveness and cancer reprogramming in CSCs or surrogate markers of therapeutic response in patients with ATRT.

STAT3 is frequently activated in various cancers and plays a crucial role in enhancing EMT and increasing invasiveness outside the CNS [43]. For example, wogonin suppresses tumor cell migration by inactivating the STAT3 signaling pathway in human alveolar cell adenocarcinomas [44]. Activation of the STAT3 pathway is required for IL-6-induced EMT in the progression of human cervical carcinomas [45]. STAT3 is also associated with sphere-forming efficiency as well as cancer stem cell-like functions in nasopharyngeal carcinoma and breast cancer [46, 47]. Induction of STAT3 signaling was associated with enhanced chemoresistance of cancer cells, while a STAT3 inhibitor was able to enhance chemosensitivity in epithelial ovarian cancer cells [48]. In ovarian cancer, high expression levels of STAT3 were shown to promote cisplatin resistance [49]. Consistent with these reports, we found that the STAT3 pathway is a key mechanism to link tumor invasion, chemoresistance and cancer stem-like properties in ATRT. In addition, EMT, the major cause of invasion, has been shown to activate chemoresistance and induce the acquisition of cancer stem-like properties [13]. However, very few reports have investigated the underlying mechanism between chemoresistance and cancer invasion with EMT-like properties in ATRT. Previous studies revealed an involvement of STAT3 in EMT through inhibition of E-cadherin expression in colorectal cancer [50]. Moreover, the STAT3/miR-34a/Snail axis promotes EMT-mediated colorectal cancer invasion and metastasis [29]. However, the exact mechanism between STAT3 and EMT is still unclear. Recent studies have shown that STAT3 directly binds to the promoter region of Beclin1 in lung cancer [51]. Here, we demonstrated that STAT3 positively promoted Snail transcription by directly binding to the Snail promoter.

Snail acts as a zinc-finger transcription factor that is essential for inducing the EMT phenotype. Previous studies reported that Snail plays important roles in the EMT phenotype of many cancer types outside of the CNS [52]. In our studies, we found an increase of Snail expression in ATRT-CisR cells compared with ATRT-Par cells. Suppression of Snail attenuated the migration and invasion in ATRT-Par/STAT3 cells. Our previous report demonstrated that ectopic expression of Snail promotes cancer stem like-cell activities by increasing IL-8 expression in human colorectal cancer cells [53]. A recent study reported that Snail directly regulates Nanog expression and enhances tumor-initiating cell characteristics [54]. In the current study, ectopic expression of Snail rescued the shSTAT3-suppressed expression of Nanog and Sox2, self-renewal ability, and tumor-initiating abilities in ATRT-CisR/sh-STAT3 cells. Based on these studies and our data, we suggest that elevated Snail expression is responsible for the increase in the EMT-like phenotype, tumor invasion, and cancer stem-like cell properties in ATRT-CisR cells.

Three ATP-binding cassette (ABC) drug transporters have been associated with drug resistance in most cancers [55], and the ABCB1 (p-glycoprotein/MDR1), ABCC1 (MRP1) and ABCC2 (MRP2) subfamilies have been shown to be involved in the development of cisplatin resistance [56–59]. Though cisplatin is one of the most commonly used chemotherapeutic drugs in most solid cancers including ATRT [36, 60], treatment with cisplatin always induces multidrug resistance along with suppressed apoptosis pathway and activated EMT [61]. Hsu et al. revealed that ectopic expression of Snail in primary head and neck cancer samples may result in cisplatin resistance and poor outcome [62]. However, the molecular mechanism of Snail-dependent induction of drug resistance in ATRT is unclear. We showed in this study that Snail overexpression not only increased cell viability after cisplatin treatment but also enhanced ABCC1 expression in ATRT-CisR/sh-STAT3 cells. Conversely, knockdown of Snail caused a distinct suppression of cell viability and attenuated the expression of ABCC1 in ATRT-Par/STAT3 cells. Taken together, these data suggest that Snail confers cisplatin resistance to ATRT-CisR cells through ABCC1.

In conclusion, our study showed that cisplatin treatment selects induced oncogenic type of drug resistance, simultaneously increased migration and invasion abilities as well as EMT-like phenotype, and promoted the acquisition of stem-like properties in ATRT cells through activation of the STAT3/Snail pathway. We suggest that Snail is a key molecule linking EMT and chemoresistance because it regulates the expression of not only EMT-related genes but also the ABCC1 transporter. We believe that STAT3/Snail signaling plays an important role in oncogenic resistance and could be a potential treatment target to inhibit ATRT invasion and to enhance the efficacy of chemotherapeutic drugs, such as cisplatin. Our study provides insight for the development of future therapies that attempt to overcome cisplatin resistance, which is frequently observed with current ATRT treatment regimens.

## METHODS

### Cell culture

All procedures of sample acquisition follow the tenets of the Declaration of Helsinki and have been approved by Institutional Review Committee at Taipei Veterans General Hospital. Between January 1998 and April 2011, a total of 32 patients with ATRT were treated at our hospital [63–65]. We had nine samples from ATRT patients. There were three females and six males as shown in Table 1. In brief, the tissues were washed in glucose containing Hanks' balanced salt solution, (HBSS; Invitrogen/Life Technologies, Carlsbad, CA, USA) three times after surgical removal of the ATRT tissues. Then, these tissues were sliced at a thickness of 300 mm and immersed in 0.1% (w/w) collagenase (Sevapharma, Prague, Czech Republic) in glucose-containing HBSS at 37°C/15 min and shaken on a rotation shaker at 125 rpm. All cells were cultured in RPMI (Gibco® 1640) with 10% FBS and antibiotics in 5% CO2 at 37°C. Analysis of cell morphology was performed using a Zeiss Axiovert 25 Phase Contrast Inverted Microscope. Digital images were captured using a Canon Power Shot G10 equipped with a Carl Zeiss 426126 lens.

### RT^2^ profiler PCR array

The Human Cell motility and Human Epithelial to Mesenchymal Transition (EMT) RT^2^ Profiler PCR Array that profiles the expression of 84 key genes was purchased from SABiosciences (Frederick, MD) and used as recommended by manufacturer. RT-PCRs were performed in 96-well plate format using the ABI 7500 FAST Real-Time PCR System. Fold changes in cell motility and EMT gene expression from denervated samples relative to control samples were calculated using the ΔΔCt method using the integrated software package for PCR Array Systems provided by the manufacturer (RT^2^ Profiler PCR Array Data Analysis Template v3.3). ΔΔCt values from each sample were normalized to three housekeeping genes that did not change across the conditions.

### Culturing cells on top of a thin or thick layer of collagen, and collection of cells from collagen matrices

These experiments used PureCor bovine collagen solution (Advance Biomatrix). For preparation of cells for seeding on top of collagen, we first seeded the cells on plastic dishes at 50% confluence. After 37°C for 10 min, we collected the cells by trypsinizing them with 0.1% trypsin in EDTA, suspended the cells at concentration of 0.4 × 10^6^ ml^−1^ and confirmed that the suspended cells were single cells by microscopic examination. A 1.6 mg ml^−1^ collagen solution (3 ml) was prepared by mixing 1.6 ml of 3 mg ml^−1^, 0.6 ml of 5x medium, PureCor bovine collagen solution, and 20 μl of 1 M NaOH, then adding water to a total volume of 3 ml. The collagen was allowed to polymerize in the tissue culture incubator at 37°C for 2 h. First, we prepared the thick collagen-coated dishes for culturing cells on top of thick collagen (2.5D) and then plated the cells on top of the collagen; an appropriate amount of serum-containing culture medium was added. By microscopic examination, we confirmed that the cells suspended in collagen were single cells and performed the cell-collagen mixture with a known amount of serum-containing medium. The experiments indicating cellular imaging in 2.5D conditions were performed using cells collected from 2.5D cultures.

### Analysis of cell morphology

We analyzed the cell morphology according to a previous report [66]. The area and the perimeter of the cells were defined by drawing around the cell shape in phase-contrast images and determined by ImageJ software. The morphology index was calculated as the perimeter^2^/4π area. We hypothesized that the ratio of a round cell is 1.0, and an elongated cell has an increased index. For every clone, the mean value of the index was determined from 200 cells.

### Quantification of the speed of motile cells by time-lapse microscopy

Time-lapse microscopic observations of cell motility were performed as described previously [67]. In a 2.5D culture, we used a 3.5-cm dish with 1 ml of the 1.6 mg ml^−1^ collagen solution, seeded 1 × 10^5^ cells on top of the collagen and then added 1.2 ml of medium. Cells were allowed to adhere for 18 h and were then observed in humidified, CO_2_-equilibrated chamber with a motorized-stage-equipped Lumascope^TM^ series for 24 h. The images were managed and reconstructed with ZEN 2009 Light Edition software. To quantify the speed of cells in the 2.5D system, we tracked the movements of individual cells with the ImageJ software Mtracking plugin analysis in a randomly selected high-power field. The cell motility speed was calculated and is presented as micrometers per minute. Each cell was tracked for identification of speed in 2.5D culture. All of the fractions of individual cells for speed identification were more than 85%. The fractions of individual cells for each clone in the speed identification experiments are described in the figure legends.

## SUPPLEMENTARY INFORMATION


