# Supplementary material for: Cisplatin-selected resistance is associated with increased motility and stem-like properties via activation of STAT3/Snail axis in atypical teratoid/rhabdoid tumor cells
Source: Oncotarget. 2015 Jan 31;6(3):1750–68. doi: 10.18632/oncotarget.2737 (PMC4359329; doi:10.18632/oncotarget.2737)
Supplement: Supplementary file 1 [file oncotarget-06-1750-s001.pdf]

SUPPLEMENTARY INFORMATION

MATERIAL AND METHOD

Cell viability assay

For evaluation of cell survival, cells were seeded on 24-well plates (Corning, Inc.) at a density of 1×10^4 cells/well, followed by the addition of methyl thiazol tetrazolium (MTT; Sigma-Aldrich) at the end of cell culture. After treatment with different concentrations of cisplatin for 24 hours, cells were incubated with MTT. The amount of MTT formazan product was determined using a microplate reader at an absorbance of 570 nm (SpectraMax 250; Molecular Devices, Sunnyvale, CA, USA). All experiments were done in triplicate, and mean values are presented.

In vitro soft agar assay

For the *In Vitro* soft agar assay, the bottom of each well (35 mm) of a six-well culture dish (Corning, Inc.) was coated with a 2-ml agar mixture [10% DMEM [volume/volume], agar (Lonza, Rockland, ME, USA)], and 0.6% fetal calf serum [weight/volume]. After the bottom layer solidified, a 2-ml top agar–medium mixture [10% DMEM [volume/volume], 0.3% fetal calf serum [weight/volume], and agar] containing 1×10^4 cells was added and incubated at 37°C/ 4 weeks. The plates were stained with 0.5 ml 0.005% crystal violet, and the number of colonies was counted using a dissecting microscope.

Quantitative real-time reverse-transcriptase (Q-PCR)

Briefly, total RNA (1 µg) from each sample was reverse-transcribed in a 20-µL reaction using 0.5 µg oligo(dT) and 200 U Superscript II RT (Invitrogen, Carlsbad, CA, USA). DNA amplification was performed in a volume of 20 µL containing 0.5 µM of each primer, 4 mM $MgCl_2$, 2 µL LightCycler™–FastStart DNA Master SYBR green I (Roche Molecular Systems, Alameda, CA, USA), and 2 µL of 1:10 diluted cDNA. Duplicate PCR reactions were heated to 95°C for 10 minutes, followed by 40 cycles of the following: denaturation at 95°C for 15 seconds, annealing at 60°C for 5 seconds, and extension at 72°C for 20 seconds. Standard curves of cycle threshold values versus template concentrations were prepared for each target gene and for internal control reference (GAPDH) in each sample. Supplemental Table 1 shows the sequences of primers used for real-time PCR experiments. Following normalization to GAPDH gene, expression levels for target gene were calculated using the comparative threshold cycle (CT) method. The Δct values were calculated according to the formula Δct=ct (gene of interest)-ct (GAPDH) in correlation analysis, and the 2-ΔΔct was calculated according to the formula ΔΔct=Δct (control group)-Δct (experimental group) for determination of relative. Data was presented as the mean ± standard deviation (S.D.) from three independent experiments.

Western blot assay

The extraction of proteins from cells and western blot analysis were performed as described [1]. Samples (15 µL) were boiled at 95°C for 5 min and separated by 10% SDS-PAGE. The proteins were wet-transferred to Hybond-ECL nitrocellulose paper (Amersham, Arlington Heights, IL, USA). Following blocking with 5% non-fat milk at room temperature for 2 h, membranes were incubated with primary antibodies (1:1000 dilution) at 4°C overnight. The following primary antibodies were used are listed in Supplemental Table 2. Then incubated with HRP-conjugated secondary antibodies (1:5000 dilution) for 2 h at room temperature. Immunoreactive protein bands were detected by the ECL detection system (Amersham Biosciences Co., Piscataway, NJ, USA).

Immunohistochemistry (IHC) staining

This research follows the tenets of the Declaration of Helsinki, and all samples were obtained after informed consent from the patients. Tumor specimens from mice or clinical human patients' tissue samples with ATRT were washed with 1X phosphate-buffered saline (PBS), fixed with 4% paraformaldehyde (Sigma), and embedded in Parafilm. All samples were spotted on glass slides for immunohistochemical staining. After deparaffinization and rehydration, tissue sections were processed with antigen retrieval by boiling in 10 mmol/L (pH 6) citrate buffer (Sigma) 3 minutes for 6 times with heating. The slides were immersed in 3% H_2O_2 for 10 minutes and washed with PBS three times. Tissue sections were blocked with serum (Vestastain Elite ABC kit, Vector Laboratories, Burlingame, CA, USA) for 30 minutes, and then incubated with the primary antibody (Supplemental Table 2) in PBS solution overnight at 4°C. Tissue slides were washed with PBS and incubated with 200x diluted biotin-labeled secondary antibody for 30 min, then incubated with streptavidin-horse radish peroxidase conjugates for 30 min, and washed with PBS three times. Tissue sections were then immersed with chromogen 3-3'-diaminobenzidine plus H_2O_2 substrate solution (Vector® DBA/Ni substrate kit, SK-4100, Vector Laboratories) for 10 minutes. Hematoxylin was applied for counter-staining (Sigma Chemical Co.,). Finally, the tumor sections were mounted with a cover slide with Gurr® (BDH Laboratory Supplies, UK) and examined under a microscope. Pathologists scoring the immunohistochemistry were blinded to the clinical data. The interpretation was done in five high-power

views for each slide, and 100 cells per view were counted for analysis. A semiquantitative scoring method that evaluates the percentage of positive tumor cells (0–100%) may provide a better understanding of the prognostic or predictive significance of these markers, such as STAT3, and Snail [2].

Overexpression and knockdown plasmid

Overexpression of STAT3 and Snail plasmid construction and DNA preparation were performed using standard procedures (Sambrook et al., 1989). The pLV vector was purchased from the National RNAi Core Facility (Academia Sinica, Taipei, Taiwan). The gene encoding human STAT3 (NM_003150) and human Snail (NM_005985) were cloned into pLV to generate the lentiviral expression vector, pLV-EF1α-IRES- STAT3 and pLV-EF1α-IRES- SnaiL. For these experiments, plasmid transfection was performed with the Trans-Messenger Reagent Kit (Qiagen, Valencia, CA).

The pLKO.1 vector was purchased from the National RNAi Core Facility (Academia Sinica, Taipei, Taiwan). The shRNA targeting human STAT3 (NM_003150, 5′-GCACAATCTACGAAGAATCAA-3′) and human Snail (NM_005985, 5′-ATGCTCATCTGGGACTCTGTC-3′) were cloned into pLKO.1 to generate the lentiviral expression vector, pLKO STAT3 and pLKO Snail. Lentivirus was produced by transfecting 293T cells at 5×10^6 cells per 10 cm plate using Lipofectamine 2000 (LF2000, Invitrogen). Supernatants were collected 48 hours after transfection and were filtered, and their viral titers were determined by FACS at 48 hours post-transduction. Subconfluent cells were infected with lentivirus at a multiplicity of infection of 5 in the presence of 8 μg/mL polybrene (Sigma-Aldrich, St Louis, MO, USA).

Cells invasion/migration analysis

On the invasion/migration assay, cells were used to prevent proliferation in the presence of 2 mM hydroxyurea in both chambers to prevent cell proliferation. The 24-well plate Transwell® system with a polycarbonate filter membrane of 8-μm pore size (Corning, NY, USA) was employed to evaluate the invasion ability of cells. The membrane was coated with Matrigel™ (BD Pharmingen, NJ, USA). The cancer cell suspensions were seeded to the upper compartment of the Transwell chamber at the cell density of 1×10^5 in 100 μl within serum-free medium. The lower chamber was filled with 10% serum. Plates were incubated for 24 hours at 37°C in 5% CO2. After 24 hours of incubation, the medium was removed and the filter membrane was fixed with 4% formalin for 1 hour. Subsequently, the remaining cells of the filter membrane faced the lower chamber was stained with Hematoxylin (Sigma-Aldrich, St Louis, MO, USA). The migrated cancer cells were then visualized and counted from 5 different visual areas of 100-fold magnification under an inverted microscope. All experiments were done in triplicate, and mean values are presented.

Expression vector delivery

Expression vectors were co-transfected into ATRT cells by Lipofectamine 2000K (Invitrogen). 50 ng of luciferase reporter vectors were cotransfected with 10 ng internal control renilla luciferase expression vectors with or without 10 ng STAT3 expression vector as indicated into each well of cell culture plates. The transfection procedures, in brief, when ATRT cells reach 30% confluent, culture medium were replenished the day before transfection. The next day, transfection cocktail of mixtures of STAT3 expression vectors and firefly reporter and renilla internal control vectors combined with lipofactamine 2000K are prepared in room temperature according to manufacturer's instructions. 20 μL of transfection cocktail are added to the culture medium for 36 h. The transfection efficiencies are monitored by examining eGFP positive cell. The transfection efficiencies are about 50%.

Constructions of expression vectors

Snail promoter region upstream 1.3 Kb region and serial deletions and site-directed mutagenesis were performed by PCR with specific primers. Promoter fragments were inserted into pGL3 reporter. 6X STAT3 RE and 6X mutSTAT3 RE were inserted into upstream region of minimal ß-actin promoter controlled firefly luciferase by inserting annealed 5′-phosphorylated 6X RE or 6X mutSTAT3 RE primer pairs. The inserted sequences were validated by sequencing. Detailed primer sequences are listed in Supplemental Table 4.

Luciferase assay

36 h after transfections, transfected cells were washed for 3 times with ice-cold PBS and total cell lysates were collected and 10 μL of total cell lysates were subjected to firefly and renilla dual luciferase assay by using Dual-light assay system (Promega). Luminescence signals were recorded by dual-mode microplate reader (Molecular Device). Relative activities were calibrated by firefly/renilla signals.

ChIP and Q-ChIP assay

For ChIP analysis, cells were co-transfected by empty vector or STAT3 expressing vectors combined with various constructs of snail promoter regions as indicated in Results. 36 h after transfection, 1×10^7 Cells were fixed by formaldehyde at room temperature for 15 min and washed 3 times by PBS. Cells were then collected in cell lysis buffer in total volumes of 200 μL and subjected to water bath-based sonication for DNA fragmentations. Immunoprecipitations were

performed by using ChIP grade anti-STAT3 antibodies (Cell Signaling) and IgG (Cell Signaling) were used as control. Immunoprecipitated DNA-protein complexes were subjected to protease K digestions and recovered DNA fragments were subjected to PCR amplifications with specific primers for various putative STAT3 binding regions. For Q-PCR analysis of endogenous STAT3 binding sites, 1 x 10^7 cells were fixed and prepared for Q-ChIP analysis by the same procedures as ChIP analysis. Endogenous putative STAT3 binding sites were amplified by specific primers sets for amplification of non-specific control, D1, D2, and D3. Q-PCR results were normalized with respective IgG internal controls. Supplemental Table 3 shows the sequences of primers used for Q-ChIP experiments.

Gene expression microarray, and similarity analysis

Total RNA was extracted from cells using TRI Reagent (Sigma, St. Louis, MO, USA) and the Qiagen RNAeasy (Qiagen, Valencia, CA, USA) column for RNA preparation. cDNA was synthesized with Superscript II RNase H-reverse transcriptase (Gibco) to generate cyanine 3 (Cy3)- and Cy5-labeled (Amersham Biosciences Co., Piscataway, NJ, USA) cDNA probes for the treated and control samples, respectively. The labeled probes were hybridized to a cDNA microarray containing 10,000 gene clone original cDNA fragments. Fluorescence intensities of Cy5 and Cy3 targets were measured and scanned separately using a GenePix 4000B Array Scanner (Axon Instruments, Burlingame, CA, USA). Then, GeneSpring GX 7.3.1 Software (Agilent Technologies, Inc., Santa Clara, CA, USA) was used for further data analysis and evaluation. GenePix Pro 3.0.5.56 software (Axon Instruments) is a powerful visualization and analysis solution designed for use with genomic expression data. The data was initially normalized by Robust Multichip Average (RMA) normalization algorithms. Statistically significant data were selected by independent sample *t*-test ($\alpha = 0.05$) between treated and control samples. The Principal component analysis (PCA) analysis and the average linkage distance were used to address the similarity between two groups of gene expression profiles as described as following. The difference in distance between two groups of sample expression profiles to a third was assessed by comparing the corresponding average linkage distances (the mean of all pairwise distances (linkages) between members of the two groups considered). The error of such a comparison was estimated by combining the standard errors (the standard deviation of pairwise linkages divided by the square root of the number of linkages) of the average linkage distances involved. Typical multidimensional scaling (MDS) was done using the standard function of the R program (http://www.r-project.org) to provide a visual impression of how the various sample groups are related.

Sphere formation assay and self-renewal assay

Tumor cells were dissociated and cultured as tumor spheres in 24-well plates (Falcon; BD) at a density of 1 x 10^5 viable cells/mL and grown in a serum-free Dulbecco's modified Eagle medium/F12 (DMEM/F12; Sigma), supplemented with N2 plus media supplement (Invitrogen), 20 ng/mL of EGF and 20 ng/mL of bFGF (Invitrogen), and penicillin/streptomycin (P/S; Invitrogen/Life Technologies). The spheroids were resuspended to form secondary and tertiary spheroids in self-renewal assay. Cells were further allowed to grow for 14 days, and the numbers of spheres were counted by microscope.

Immunofluorescence staining

The cells were cultured on chamber slides for 24 h, and then washed four times with PBS. Then the cells were fixed with 4% paraformaldehyde for 20 minutes and permeabilized with 0.3% TritonX-100 for 10 minutes. After blocked with goat serum for two hours at room temperature, cells were incubated with antibodies against E-cadherin, N-cadherin (1:100 dilution) at 4°C overnight. Slides were washed four times with PBS and incubated with Alexa Fluor488 or Alexa Fluor 594-conjugated secondary antibodies (1:1000 dilution) for 1 h at room temperature. Nuclei were stained with DAPI (10 μg/ml) for10 min. Samples were examined with Confocal Laser Scanning Microscopy (Zeiss) to analyze expression of E-cadherin and N-cadherin.

Annexin V apoptosis staining

Apoptotic cell was measured by flow cytometry using FITC annexin V and propidium iodine (PI) apoptosis detection kit I from BD Pharmingen (San Jose, CA). After incubation with FITC annexin V and PI at room temparature in the dark for 10 min, cells were subjected to BD Calibur cytometer analysis at the OHSU flow cytometry core. Unstained cells, cells stained with FITC annexin V (no PI) and with PI (no FITC annexin V) were used to set up compensation and quadrants for analysis

Cisplatin treatment and clonogenic assay

Briefly, cells in the control and cisplatin treated groups were exposed to different cisplatin dosages (0, 1, 3, 5, and 10 μg/ml). After incubating for 24 hours, colonies (> 50 cells per colony) were fixed and stained for 20 minutes with a solution containing crystal violet and methanol. Cell survival was determined by a colony formation assay. Plating efficiency (PE) and survival fraction (SF) were calculated as follows: PE = (colony number/number of inoculated cells) × 100%; SF = colonies counted/ (cells seeded × [PE/100]).

In vivo RFP imaging of tumor growth

All procedures involving animals were in accordance with the institutional animal welfare guidelines and approved animal protocol of the Taipei Veterans General Hospital. Female SCID, 7–8 weeks of age, were obtained from the National Laboratory Animal Center (Taipei, Taiwan). SCID was intracranial (IC) injected with ATRT cells in 20 ml PBS. For IC, the skulls of the mice were immobilized in a stereotaxic apparatus and a ~1.5-mm hole was created in the cranium by rotating fine handheld tweezers in a circular motion; ATRT cells were re-suspended in 10 ml of phosphate buffered saline (PBS), in aliquots of 1 x 10^5 cells. Following administration of general anesthesia, these aliquots were injected stereotactically into the frontal cortex of 7- to 8-week-old NOD-SCID mice. The injection coordinates were 2 mm to the right of the midline, 2 mm anterior to the coronal suture, and 3.5 mm deep. The tumor size was assessed by 3T-MRI. *In vivo* RFP imaging was performed using an illuminating device (LT-9500 Illumatool TLS equipped with a excitation illuminating source [470 nm] and filter plate [515 nm]). ATRT-CisR cells were re-suspended in 10 ml of phosphate buffered saline (PBS), in aliquots of 1 x 10^5 cells.

3T-MRI

Tumor volume was calculated at weekly intervals performing a 3T-MR imaging Biospect system (Bruker, Ettlingen, Germany) with a miniquadrature coil for radiofrequency transmission and reception of 3T-MR imaging signals. The volume was measured [(length x $width^2$)/2] and therefore analyzed performing Image-Pro Plus software (Media Cybernetics, Rockville, MD, USA) [3, 4].

Statistical analysis

Results are reported as mean ± standard deviation values. Statistical analysis was performed using two-tailed independent Student's *t*-test or a one-way or two-way analysis of variance (ANOVA) followed by Turkey's test, as appropriate. Survival was estimated by using the Kaplan-Meier method and was compared between groups with the log-rank test. All data were derived from independent experiments, and the cells were not pooled across experiments. $P < 0.05$ was considered to be statistically significant.

REFERENCES

1. Kao CL, Chiou SH, Chen YJ, Singh S, Lin HT, Liu RS, Lo CW, Yang CC, Chi CW, Lee CH, Wong TT. Increased expression of osteopontin gene in atypical teratoid/rhabdoid tumor of the central nervous system. Modern pathology : an official journal of the United States and Canadian Academy of Pathology, Inc. 2005; 18:769–778.
2. Zlobec I, Steele R, Michel RP, Compton CC, Lugli A, Jass JR. Scoring of p53, VEGF, Bcl-2 and APAF-1 immunohistochemistry and interobserver reliability in colorectal cancer. Modern pathology : an official journal of the United States and Canadian Academy of Pathology, Inc. 2006; 19: 1236–1242
3. Lee YY, Yang YP, Huang MC, Wang ML, Yen SH, Huang, PI, Chen YW, Chiou SH, Lan YT, Ma HI, Shih YH, Chen MT. MicroRNA142-3p promotes tumor-initiating and radioresistant properties in malignant pediatric brain tumors. Cell transplantation. 2014; 23: 669–690
4. Chiou GY, Chien CS, Wang ML, Chen MT, Yang YP, Yu YL, Chien Y, Chang YC, Shen CC, Chio CC, Lu KH, Ma HI, Chen KH, Liu DM, Miller SA, Chen YW. et al.Epigenetic regulation of the miR142-3p/interleukin-6 circuit in glioblastoma. Molecular cell. 2013; 52: 693– 706

SUPPLEMENTARY FIGURES AND TABLES

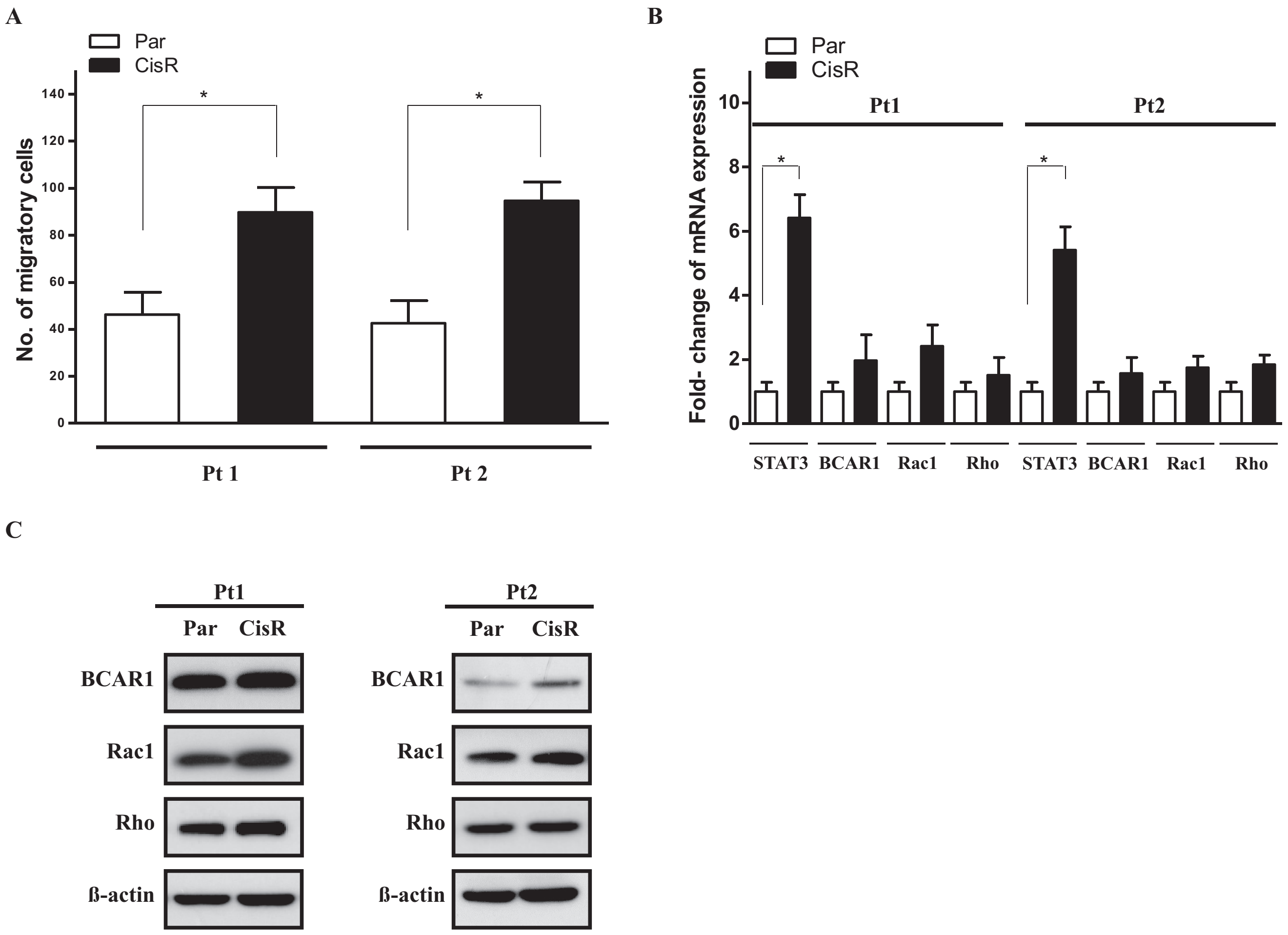

Supplementary Figure 1: Cisplatin-resistant ATRT cells have a more migratory phenotype and increased expression of STAT3. (A) Pt1-Par, Pt2-Par, Pt1-CisR, and Pt2-CisR cells were subjected to migration assay. *$P < 0.01$ by Student's *t*-test. **(B)** A qPCR analysis of the protein level of the motility-related genes of STAT3, BCAR1, Rac1, and Rho. **(C)** Western blot analysis of the motility-related genes of STAT3, BCAR1, Rac1, and Rho. The data shown are the mean ± SD of three independent experiments.

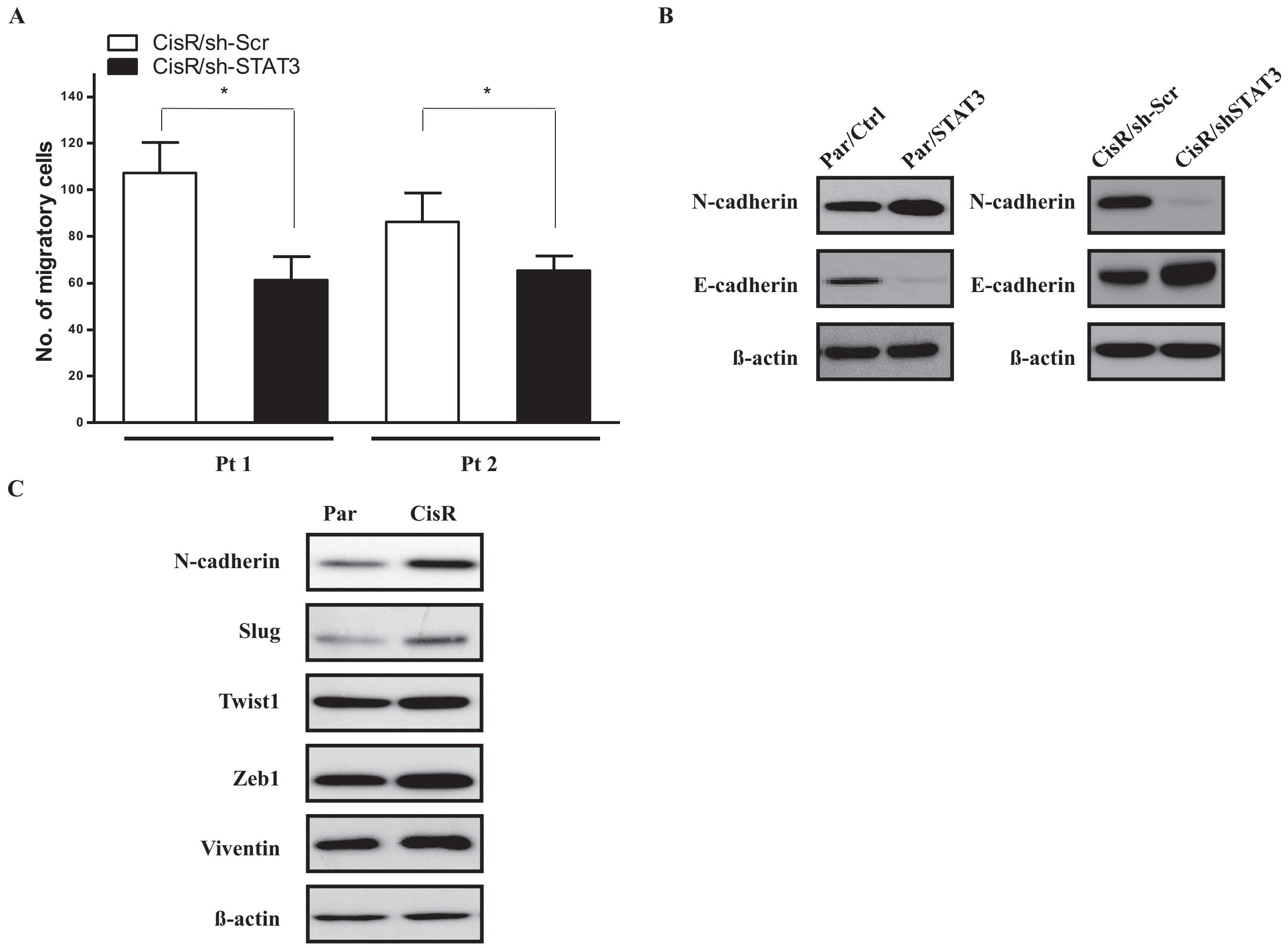

Supplementary Figure 2: EMT-related factors are downstream effectors of STAT3-induced tumor invasion. (A) Pt1-CisR cells transfected with the scrambled shRNA control vector (Pt1-CisR/sh-Scr), Pt1-CisR cells transfected with the sh-STAT3 vector (Pt1-CisR/sh-STAT3), Pt2- CisR cells transfected with the scrambled shRNA control vector (Pt2-CisR/sh-Scr), and Pt2- CisR cells transfected with the sh-STAT3 vector (Pt2-CisR/sh-STAT3) were subjected to migration assay. *$P < 0.01$ by Student's *t*-test. **(B)** Western blot analysis of N-cadherin and E-cadherin were analyzed in Par/Ctrl, Par/STAT3, CisR/sh-Scr, and CisR/sh-STAT3 cells. **(C)** Western blot analysis of the activator of EMT, such as N-cadherin, Snail, Slug, Twist1, Zeb1 and Vimentin. The data shown are the mean ± SD of three independent experiments.

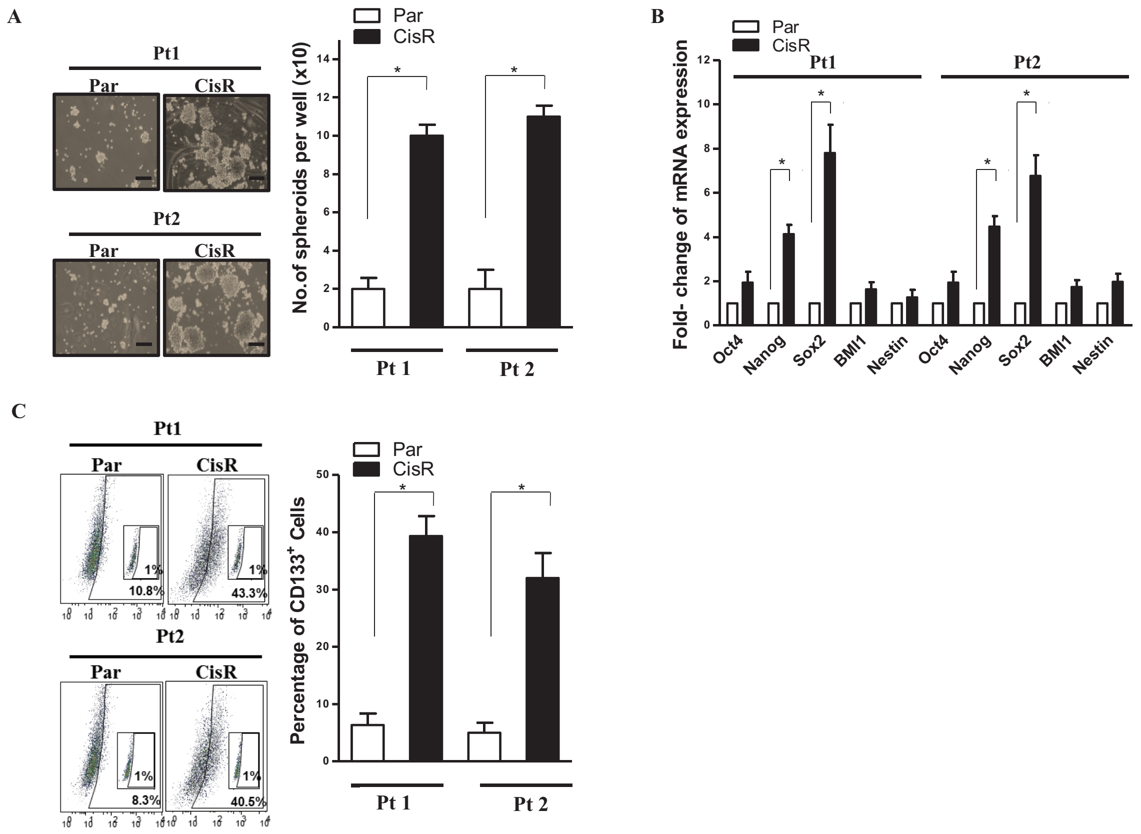

Supplementary Figure 3: The STAT3/Snail axis acquires the stemness and tumor-initiating capacities in ATRT-CisR cells. **(A)** In sphere-forming assay, ATRT-CisR cells acquire higher sphere-forming numbers than ATRT-Par cells. Scale bars, 50 μm. $*P < 0.01$ by Student's *t*-test. **(B)** A qPCR analysis of Oct-4, Nanog, Sox2, Bmi-1, and Nestin in ATRT-Par cells compared with ATRT-CisR cells. $*P < 0.01$ by Student's *t*-test. **(C)** The presence of $CD133^+$ positive cells of ATRT-Par cells compared with that of ATRT-CisR cells by flow cytometry. $*P < 0.01$ by Student's *t*-test. The data shown are the mean ± SD of three independent experiments.

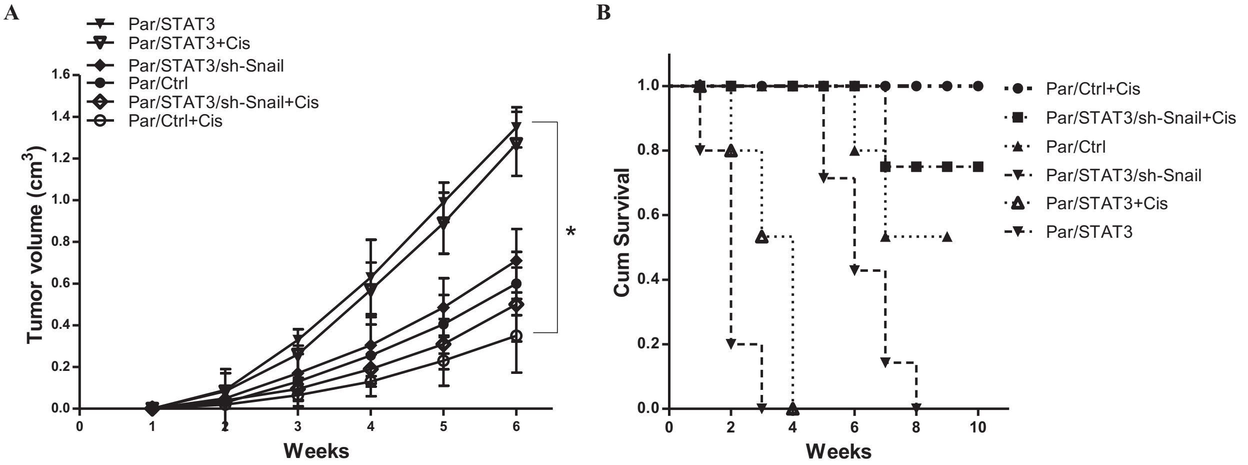

Supplementary Figure 4: STAT3/Snail axis silencing increases the synergistic effects with chemosensitivity and prolongs the survival of ATRT-CisR *in vivo*. ATRT-Par were intracranially transplanted into NOD-SCID mice, and six mice in each group ($n = 6$ in each group; total 36 mice). **(A)** Tumor volumes in ATRT-Par transplanted mice treated with vector control (Ctrl) combined with cisplatin (3 μg/ml) treatment were significantly smaller than those receiving different protocol. $*P < 0.01$ by Student's *t*-test. **(B)** Kaplan-Meier survival analysis further revealed that the mean survival rate for animals injected with ATRT-Par treated with indicated treatments. $*P < 0.01$ by Student's *t*-test. The data shown are the mean ± SD of three independent experiments.

Supplementary Table 1: The sequences of the primers for quantitative RT-PCR

Gene (Accession No.)	Primer Sequence (5′ to 3′)	Product size (bp)	Tm (°C)
STAT3 (NM_003150)	F: AGCAGCACCTTCAGGATGTC<br>R: GCATCTTCTGCCTGGTCACT	168	60
Snail (NM_005985)	F: CGAGCTGCAGGACTCTAAT<br>R: CCACTGTCCTCATCTGACA	231	55
BRCA1 (NM_007294)	F: TGTGAGGCACCTGTGGTGA<br>R: CAGCTCCTGGCACTGGTAGAG	69	55
Rac1 (NM_006908)	F: CACGATCGAGAAACTGAAGGA<br>R: AGCAGGCATTTTCTCTTCCTC	201	58
Rho (NM_000539)	F: GAAGCCACCTGCTCTTTTGC<br>R: CAAGGAAGGTAGGCCCAGTG	174	55
N-cadherin (NM_001792)	F: CCACGCCGAGCCCCAGTATC<br>R: CCCCCAGTCGTTCAGGTAATCA	232	61
Slug (NM_003068)	F: GTGATTATTTCCCCGTATCTCTAT<br>R: CAATGGCATGGGGGTCTGAAAG	292	55
Twist1 (NM_000474)	F: GGGAGTCCGCAGTCTTACGA<br>R: AGACCGAGAAGGCGTAGCTG	277	61
Zeb1 (NM_030751)	F: ACTGCTGGGAGGATGACAGA<br>R: ATCCTGCTTCATCTGCCTGA	72	55
Vimentin (NM_003380)	F: GCAATCTTTCAGACAGGATGTTGAC<br>R: GATTTCCTCTTCGTGGAGTTTCTTC	118	59
Oct-4 (NM_002701)	F: TGTGGACCTCAGGTTGGACT<br>R: CTTCTGCAGGGCTTTCATGT	207	58
Nanog (NM_024865)	F: TCTTCCTACCACCAGGGATGC<br>R: CACTGGCAGGAGAATTTGGC	250	59
Sox2 (NM_003106)	F: CGAGTGGAAACTTTTGTCGGA<br>R: TGTGCAGCGCTCGCAG	74	58
Nestin (NM_006617)	F: AGGAGGAGTTGGGTTCTG<br>R: GGAGTGGAGTCTGGAAGG	112	55
Bmi1 (NM_ 005180)	F:AAATGCTGGAGAACTGGAAAG<br>R:CTGTGGATGAGGAGACTGC	124	57
GAPDH (NM_002046)	F: CATCATCCCTGCCTCTACTG<br>R: GCCTGCTTCACCACCTTC	180	58

Bp, base pairs; Sox2, sex-determining region; GAPDH, glyceraldehyde 3-phosphate dehydrogenase.

Supplementary Table 2: List of proteins tested by antibodies

Protein	Assay	Antibody	Origin	Dilution	Incubation period
STAT3	WB<br>IF<br>IHC	mmab	#9139, Cell Signaling, Inc	1:1000<br>1:1000<br>1:500	overnight
p-STAT3	WB	mmab	#4113, Cell Signaling, Inc	1:1000	overnight
Snail	WB<br>IHC	rpab	Ab180714, Abcam, Inc	1:1000<br>1:200	overnight
BCAR1	WB	rpab	Ab80016, Abcam, Inc	1:1000	overnight
Rac1	WB	mmab	Ab33186, Abcam, Inc	1:1000	overnight.
Rho	WB	rmab	Ab17732, Abcam, Inc	1:2000	overnight
N-cadherin	WB<br>IF	rpab	Ab18203, Abcam, Inc	1:1000<br>1:200	overnight
E-cadherin	WB<br>IF	mmab	Ab76055, Abcam, Inc	1:1000<br>1:200	overnight
Slug	WB	rpab	Ab38551, Abcam, Inc	1:1000	overnight
Twist1	WB	rpab	#4119, Cell Signaling, Inc	1:1000	overnight
Zeb1	WB	mmab	Ab180905, Abcam, Inc	1:2000	overnight
Vimentin	WB	rpab	#4745, Cell Signaling, Inc	1:1000	overnight
Fibronectin	IF	rpab	Ab2413, Abcam, Inc	1:200	overnight
ß-actin	WB	mmab	Ab3280, Abcam, Inc	1:10000	2hrs

Abbreviations: WB, Western blot; mmab, mouse monoclonal antibody; rmab, rabbit monoclonal antibody; rpab, rabbit polyclonal antibody; IF, immunofluorescence; IHC, Immunohistochemistry.

Supplementary Table 3: Primers for Snail promoter constructions, ChIP and Q-ChIP

Primers for Snail promoter constructions, specific PCR, ChIP		
Snail	Snail Full F	5′-AGGTGGAAACAAGAACGGTGAGACC-3′
	Snail Full R	5′-TTCGCGCAGCAGTAGCGCAGAAGAA-3′
	Snail -D1 F	5′-GTCCCTCCTCAGCTGAAAATCCTTC-3′
	Snail -D2 F	5′-CCATCCCACCCCATCCCTGGAAGCT-3′
	Snail -D3 F	5′-CTCCCTCACTGGACCAGAAGCTACC-3′
	Mut Snail F	5′-CTAGGAGTTACTCTGGGGCAGTTGC-3′
	Mut Snail R	5′-GCAACTGCCCCAGAGTAACTCCTAG-3′
ChIP and Q-ChIP for Snail	–909~–900 F	5′-AGTCCAAACTCCTACGAGGCCCTGG-3′
	–909~–900 R	5′-AAAAGGCCCGAGGGAAAGAAGTGGC-3′
	–1011~–1003 F	5′-CTAACCAGGTCCCTCCTCAGCTGAA-3′
	–1011~–1003 R	5′-CTCCTAGAGAGCAGCTTCCAGGGAT-3′
	–1077~–1070 F	5′-TATGGAGCCGTGTTACAGCCTTTAG-3′
	v1077~–1070 R	5′-ACCGAAGGATTTTCAGCTGAGGAGG-3′
	N. C. F	5′-ATGCATTCAGTCAGGGCCCCTATGG-3′
	N. C. R	5′-GGAGGGACCTGGTTAGAGTTTCGTT-3′

ChIP: chromatin immunoprecipitation. N.C: Non-specific control region.

Supplementary Table 4: Primers for 6xRE STAT3 binding sites reporter construction

Primers for 6xRE STAT3 binding sites reporter construction		
6xRE STAT3	Forward synthesized 5′-phosphorylated	5′-pTTACTCTGAAAATTACTCTGAAAATTACTCTGAAAAT TACTCTGAAAA TTACTCTGAAAATTACTCTGAAAA-3′
	Reverse synthesized 5′-phosphorylated	5′-pTTTTCAGAGTAATTTTCAGAGTAATTTTCAGAGTAA TTTTCAGAGTAATTTTCAGAGTAATTTTCAGAGTAA-3′
Mutated 6xRE STAT3	Forward synthesized 5′-phosphorylated	5′-pTTACTCTGGGAATTACTCTGGGAATTACTCTGGGAAT TACTCTGGGAA TTACTCTGGGAATTACTCTGGGAA-3′
	Reverse synthesized 5′-phosphorylated	5′-pTTCCCAGAGTAATTCCCAGAGTAATTCCCAGAGTAAT TCCCAGAGTAATTCCCAGAGTAATTCCCAGAGTAA-3′
